# Preclinical screening models in anticancer drug development: strengths, limitations, and translational challenges

**DOI:** 10.3389/fphar.2026.1822764

**Published:** 2026-05-11

**Authors:** Rishikesh Yadav, Shivam Singh, Sandeep Kumar, Arindam Kolay, Himanshu Sharma, Rashmi Dorai, Ashish Kumar Sharma, Ravindra Pal Singh, Meena K. Yadav

**Affiliations:** 1 Department of Pharmacology, Nims Institute of Pharmacy, Nims University Rajasthan, Jaipur, India; 2 Department of Pharmaceutics, Chitkara College of Pharmacy, Chitkara University, Rajpura, India; 3 Department of Pharmaceutics, Nims Institute of Pharmacy, Nims University Rajasthan, Jaipur, India; 4 Department of Pharmaceutical Chemistry, Nims Institute of Pharmacy, Nims University Rajasthan, Jaipur, India

**Keywords:** angiogenesis, anti-cancer, anticancer drug development, cytotoxic, drug development, organoids, screening models, translational challenges

## Abstract

Cancer is one of the leading causes of morbidity and mortality worldwide, accounting for a considerable global health burden and approximately 10 million deaths every year. There have been monumental breakthroughs in cancer research; but translating promising preclinical data into successful evolving drug therapies continues to have an enormous challenge. This challenge is further emphasised by the very high rate of attrition throughout clinical development, confirming the inadequacies of existing predictive screening platforms and emphasising the need for more predictive preclinical models. Therefore, this paper provides an overview of the various major types of preclinical models used to evaluate anticancer drugs, along with their respective strengths, weaknesses, and translational relevance. Classical *in vitro* cytotoxicity screens provide an essential element to early phase high-throughput cytotoxicity evaluations. More sophisticated 3D and 4D *in vitro* tumour models, patient-derived organoids, and organ-on-chip technologies allow for improved modelling of tumour structure and architecture, microenvironmental complexity, and drug penetration dynamics, improving accuracy in predicting *in vivo* activity of agents being screened *in vitro*. Further, *in vivo* model systems, provide essential *in vivo* data to dissect and understand the biology of tumours, pharmacokinetics, toxicity, and therapeutic efficacy of agents used to treat cancer. Rational drug discovery will be further enhanced through increasingly available emerging technologies such as bioinformatics, molecular docking, genomics and omics-based methods. Although traditional animal and cell line models are needed in drug discovery; the shortcomings of these systems, in their inability to reproduce the full complexity of human tumours means they account for a large proportion of translational failures. This comprehensive review shows how developing multi-faceted, human-comparable and technologically sophisticated screening platforms for test drugs will increase predictivity, speed up drug development for cancer treatment and improve clinical benefit from tested drugs.

## Graphical Abstract

**Figure:**
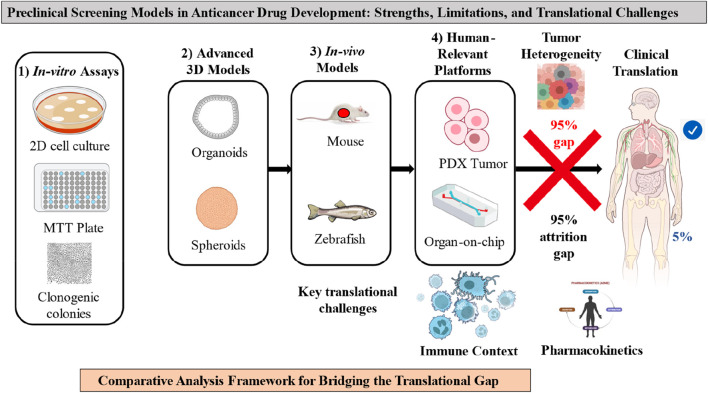
Comparative Analysis Framework for Bridging the Translational Gap.

## 1 Introduction

Cancer stands as one of the greatest global health, social, and economic challenges of the 21st century. Globally, it accounts for nearly one in every six deaths and contributes to almost one in four deaths caused by NCDs. Alarmingly, cancer is responsible for about 30% of premature deaths from NCDs in people aged 30–69 years. Across 177 of 183 countries, it ranks among the top three causes of death in this age group (Bray et al., 2021; Irie and Kuratani, 2011). Beyond limiting improvements in life expectancy, cancer also places a heavy burden on society and the economy. The extent of this impact differs depending on the type of cancer, the region, and even between men and women (Chen et al., 2023).

A recent study highlighted the severe consequences of cancer-related deaths among women (Guida et al., 2022). It is estimated that in 2020, one million children worldwide became maternal orphans as their mothers succumbed to cancer. What is alarming is that almost half of these maternal orphanhood’s were associated with deaths due to breast and cervical cancers. New anti-cancer drugs are being developed either through the use of intensive drug-screening strategies or through the design of molecules that target particular pathways in tumours based on biological knowledge. These new drugs are expected to be more effective and have less toxicity than the conventional therapies of chemotherapy and radiation.

Nevertheless, assessing and eventually using these new agents in everyday medical practice poses significant challenges for research at all stages: basic, preclinical, and clinical. Rodent models fulfil regulatory requirements but exhibit only 3.4% predictivity for Phase I approval despite promising efficacy data. Mouse cytochrome P450 enzymes metabolize drugs 10× faster than humans, causing 70% pharmacokinetic discrepancies. Immunotherapies achieve 80%–100% response rates in syngeneic models versus 20% clinical ORR due to Fc receptor and MHC mismatches. Homogeneous tumours fail to capture human intratumor heterogeneity (>10^6^ clones) and TME-mediated immune exclusion. These species-specific limitations explain the 95% preclinical-clinical attrition rate, underscoring the need for human-relevant validation platforms beyond traditional animal testing (Zips et al., 2005). The mechanisms underlying this complex disease remain incompletely understood. However, activation of proto-oncogenes and suppression of tumour-suppressor genes are recognized as major contributors to cancer development.

Furthermore, most drugs aimed at preventing cancer focus on angiogenesis, an important aspect of the disease’s mechanisms. A variety of molecules collaborate to disturb the well-organised process of angiogenesis. In lung adenocarcinoma, the main mediator of angiogenesis linked to tumours is VEGF, which promotes the growth and dissemination of tumours (Fukumura et al., 1998). Specifically, VEGF-A is considered the principal angiogenic factor in human lung cancer.

Many tumours exhibit elevated levels of pituitary tumour-transforming gene-1 (PTTG1) (Vlotides et al., 2007). Downregulation of FOXO1 has been associated with promotion of cell proliferation in cervical cancer (Prasad et al., 2014). Ongoing research continues to identify new molecular targets, making anticancer drug development more efficient. Cancer cells can spread through the bloodstream to nearby or distant sites. In advanced stages, death may result from ineffective treatment, misdiagnosis, or widespread metastasis. One reason for the failure of treatment is the development of resistance to cancer-fighting medications. Common anticancer drugs are often associated with severe side effects, including hair loss, bone-marrow suppression, and nausea. Since many cancer therapies remain in developmental stages, they are also prohibitively expensive for most patients. Thus, rapidly identifying effective treatment strategies for human cancers remains a major challenge. Rapid screening of large numbers of compounds is therefore crucial.

*In vitro* assays, such as ligand-binding or inhibition studies, are commonly employed for systematic evaluation of anticancer drugs. This review will cover the benefits and drawbacks of different screening methods used in the discovery of anticancer medications. Recent challenges with anticancer drug screening have persisted despite tremendous scientific advancements, especially in converting encouraging lab results into successful clinical treatments. Although new high-throughput screening platforms, organoid and spheroid models, and computational approaches have revolutionized *in vitro* testing, these systems often fail to fully reproduce the complexity of the tumour microenvironment, immune interactions, and metastatic behaviour observed *in vivo*. There is an extensive translational gap from preclinical to clinical due to several variables affecting cancer drug development, resulting in only 3%–8% of oncology drugs receiving approval. Some of these variables include tumour heterogeneity, lack of microenvironment complexity, variability in immune systems, and interspecies pharmacokinetic differences. Thus, to improve clinical translation, it will be necessary to develop sophisticated experimental models such as 3D cell culture systems, patient-derived organoids, and new computational techniques.

Although there have been many reviews published about anticancer drug discovery and screening methods, this review is different and timely in that it will focus on the continuum of anticancer drug development process from *in vitro* drug screening to eventual clinical relevance. Past reports have primarily focused on either molecular target, the high-throughput screening technology used, or animal models alone. Conversely, this review will consider traditional cell-based assay systems, new high-tech three-dimensional culture systems (e.g., organoid and spheroid platforms), and the various approaches to validate these models *in vivo* and with the use of new computational tools in an integrated way along the entire continuum from drug development through all stages of the drug approval process. This review will place a particular focus on discussing the predictive value, limitations, willingness to scale, and level of clinical relevance of different screening platforms, as they relate to modern oncology drug development. The review will illustrate the continuing barriers to translating laboratory data into successful patient outcomes, as well as describe emerging methods that could help overcome this barrier, such as the integration of physiologically relevant models and precision-targeted drug screening platforms. Overall, our intent with this review is to present current technically advanced methodologies that can then be related to current translational barriers in the hopes of providing new ideas for accelerating the development of new safe, effective and clinically relevant anticancer drugs.

## 2 Screening models for anti-cancer drug evaluation

### 2.1 *In vitro* methods

**FIGURE 1:**
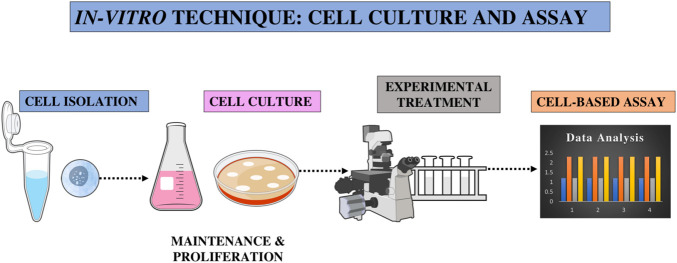
Overview of *in vitro* experimental procedure: Cell Isolation, Culture, Treatment, and Assay.

Before evaluating a potential chemotherapy agent in living organisms, it is generally preferable to begin testing under controlled laboratory conditions. This approach helps researchers obtain preliminary information about the drug’s effectiveness and safety before moving into more complex and ethically sensitive animal studies. Although animal experiments are considered to be more accurate since they can better simulate the human biological response, laboratory models of experiments have a number of advantages that make them extremely useful in the early stages of research. Laboratory models of experiments are much less time-consuming, allowing experiments to be carried out and repeated within a short period of time. They are also much more cost-effective, reducing the financial burden of large-scale experiments on animals. Another advantage is that a small amount of the test compound is required, allowing a large number of potential drugs to be screened quickly and efficiently. Moreover, laboratory models of experiments are easier to handle and control, as they eliminate variability and allow consistent experimental conditions. All these advantages make laboratory testing a vital step in the drug development process, allowing scientists to select the most promising candidates for further testing on living organisms has been depicted in Figure 1. In addition, controlled parameters such as pH, temperature, humidity, and oxygen-carbon dioxide ratio also promote the growth of *in vitro* cultures, resulting in the production of consistent cell populations and reducing the chances.

These methods may lead to false -positive or false-negative outcomes. Some substances that show activity *in vitro* fail to demonstrate similar effects in living organisms. A false-negative result occurs when a potentially active compound is mistakenly excluded from further stages of the screening process. This may occur because the compound’s transport or metabolism *in vivo* enhances its activity, which cannot be observed *in vitro*. Another limitation is that pharmacokinetic properties of drugs cannot be accurately evaluated using *in vitro* methods. Additionally, the structure of solid tumours *in vivo* is very different from that of cells grown in monolayer cultures or suspended *in vitro* (Kruh and Belinsky, 2003). Cell lines provide reproducibility and scalability for initial hit identification via MTT/SRB assays, processing thousands of compounds daily. However, they fail to recapitulate stroma interactions, hypoxia gradients, ECM stiffness, and metastatic niches critical for clinical response. Genetic drift from prolonged culture creates hypermutated profiles unlike primary tumours. This disconnect explains the 95% attrition rate 2D IC_50_ values correlate poorly with patient outcomes, necessitating validation in 3D organoids (see Table 1).

**TABLE 1:** Comparative predictive value of *in-vitro* assay.

Assay	Predictive accuracy	Throughput	Cost	Clinical correlation	Translational success rate	References
MTT (Tetrazolium)	Low (metabolic bias)	High (96-well)	Low	Poor (false positives)	∼5–10% overall preclinical-clinical	Banasiak et al. (1999), Hutchinson and Kirk (2011)
SRB	Moderate (protein-based)	High	Low	Fair (biomass measure)	∼5–10%	Banasiak et al. (1999), Hutchinson and Kirk (2011)
^3^H-thymidine	High (DNA synthesis)	Low	High	Moderate (proliferation)	20%–30%	Moschopoulos et al. (2025), Long et al. (2022)
Dye exclusion	Very low (membrane only)	Low	Very low	Poor (late apoptosis)	<5%	Hutchinson and Kirk (2011), Strober (1997)
Clonogenic	High (reproductive death)	Very low	Moderate	Strong (r = 0.59–0.82)	50%–60%	Scholz et al. (1990)

The ideal characteristics of an *in vitro* screening method include being quick, cost-effective, reproducible, simple, and accurate. It is important that the cell lines mirror those found in clinical situations as closely as possible. Comparisons should be made between the medication concentrations tested *in vitro* and those expected for actual treatment *in vivo*. The test should efficiently process a large number of samples automatically. Additionally, gathering data should be easy, clear, and practical.

At this time, a system like that does not exist. A screening test tries to find out if a medication can kill cells, but it is also important to identify which cells are dividing and which are not, such as inactive cells that are dead or near death due to apoptosis. Comparative overview of *in vitro* cell-based assay methods, their measured cellular properties, advantages, limitations, and translational relevance in preclinical drug screening has been shown in Table 2 (Thayer et al., 1971). A testing method is used to find out if a drug can kill cells, but it must also identify which cells are actively dividing and which ones are not (dormant cells that are either dead or in the process of dying (apoptosis).

**TABLE 2:** Comparative overview of *in vitro* cell-based assay methods, their measured cellular properties, advantages, limitations, and translational relevance in preclinical drug screening.

S. No	Assay methods	Biological parameter measured	Key advantages	Key limitations	Biological parameter measured/Translational relevance
1	Assay for Tetrazolium Salt (MTT)	Mitochondrial Metabolic Activity/Viability	Fast and high-throughput for initial screening	Indirect measure (enzyme activity only); prone to chemical interference; requires extra solubilization step	Reflects cell metabolic competence and viability; widely used for preliminary cytotoxicity screening in anticancer and neuroprotective studies
2	Sulphorhodamine B (SRB) test	Total Cellular Protein Mass/Cell Density	Stable, reproducible endpoint; measures total biomass	Indirect measure of cell number; not suitable for real-time studies (cells fixed)	Indicates overall biomass and cell growth inhibition; useful for drug-induced antiproliferative screening
3	^3^H-thymidine incorporation assay	DNA Content/Synthesis/Cell Proliferation	Direct measurement of cells undergoing DNA synthesis (S-phase); non-radioactive methods highly sensitive	Radioactivity issues (thymidine); requires complex equipment; cannot distinguish proliferation from DNA repair	Measures DNA replication and proliferative index; highly relevant for tumour growth kinetics and cell-cycle studies
4	Tests for dye exclusion (e.g., Trypan Blue, Propidium Iodide)	Membrane Integrity/Permeability	Simple, quick, and inexpensive live/dead count	Low throughput; subjective manual counting; detects late-stage cell death	Assesses cell membrane damage and necrotic/apoptotic death; useful for acute cytotoxicity assessment
5	Clonogenic assay	Reproductive Integrity/Clonogenicity	Gold standard for reproductive cell death; biologically most relevant	Very slow (weeks); low throughput; limited to colony-forming cells	Measures long-term survival and proliferative recovery capacity; strong translational relevance in oncology and radiobiology
6	Cell counting test	Cell Number/Growth Rate	Provides absolute cell number; easy to interpret	Manual counting is laborious and error-prone; automated systems costly	Reflects absolute growth kinetics and proliferation rate; important for dose-response normalization and growth curve analysis
7	Western blotting, ELISA, or Reporter Assays	Molecular Target Expression/Signaling Activity	Highly specific (Western blot); quantitative (ELISA); functional pathway analysis (reporter assays)	Low throughput (Western blot); time-consuming; indirect functional inference	Measures specific protein expression, signaling pathways, and mechanistic biomarkers; highly valuable for translational target validation and mechanism-of-action studies

#### 2.1.1 Tetrazolium salt assay (Microculture Tetrazolium Test or MTT)

**FIGURE 2:**
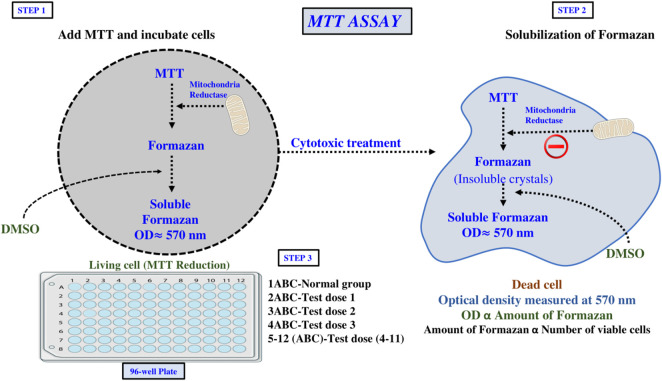
Tetrazolium salt assay (microculture tetrazolium test or MTT).

A commonly used *in vitro* method for assessing possible anticancer drugs is the MTT assay. While there are numerous staining techniques for evaluating cell viability, the majority of them require washing procedures, which add to sample variability and processing time. In contrast, multi-well plate spectrophotometers allow for the quick and accurate examination of a large number of samples. For live cells, a colourless substrate that living cells can transform into a coloured result is suitable for a colorimetric assay; non-viable cells and culture medium should remain inactive. This idea is applied in the MTT assay, which detects the presence of metabolically active cells via a colorimetric shift (Mosmann, 1983). Through the action of mitochondrial dehydrogenase enzymes, the yellow tetrazolium salt MTT can be reduced into an insoluble blue formazan product, which is the basis for the MTT assay as shown in Figure 2 (Prakash et al., 1999).

However, there are some limitations associated with this assay. In addition, one of the limitations to this assay is that it may cause the cell to appear metabolically active but not proliferating due to the fact that it cannot distinguish between reduced cell mitosis and decreased metabolic function. Use of the mitochondrial function inhibitors would also affect this assay and would affect the results particularly for short incubation times. A vehicle control, which in most cases is DMSO, should be taken into consideration since it can affect cell survival and can have an effect on the assay results. As previously mentioned, even though there are limitations associated with this assay it remains a standard method for the initial assessment of drug cytotoxicity and antiproliferative potential and is still frequently used (Alley et al., 1988).

#### 2.1.2 Sulphorhodamine B assay

The SRB assay is used to quantify total protein in all the cultures and is expected to be proportional to the number of cells in the culture. The SRB assay is a protein-based assay that provides a quantitative measure of the viability of cells in both adherent and suspension cultures. The SRB assay uses SRB, a bright pink anionic dye, that binds to the basic amino acid residues within cellular proteins. Following the removal of unbound dye, the protein-bound SRB is made soluble by the addition of Tris base to the media, and the optical density is measured using a 96-well microtiter plate reader. The dead cells have either lysed or been lost prior to analysis, and thus the amount of protein-bound SRB indicates the total amount of protein in the surviving cells after treatment with drugs. The SRB assay provides a higher degree of reproducibility, screen capacity and quality control compared to the tetrazolium-based assay. The MTT assay has more laboratory labour and is a longer process than the SRB assay; it has the additional disadvantage that residual protein from non-viable or non-dividing cells can interfere with measurement of protein, thus possibly overestimating cell mass (Skehan et al., 1990).

#### 2.1.3 ^3^H-thymidine incorporation assay

A radio-labelled precursor (^3^H-thymidine) is introduced during the last 48 h of the assay to mark proliferating cells after cancer cell suspensions have been continually exposed to the medication for 5 days. ^3^H-thymidine will be incorporated into the DNA of the replicating cells, and this can be detected by liquid scintillation counting or autoradiography. Although autoradiographic measurement of ^3^H-thymidine takes a lot of time, it offers insights into the dynamics of cancer growth. The resulting DNA histograms may reveal details about the cell’s ploidy status. Cells that have actively replicated DNA and are therefore viable are examined in this experiment. In this instance, dead or non-replicating cells will not be included. Both suspension and adherent cell lines can be subjected to the assay. The test has the disadvantage of being labour-intensive and utilizing radioactivity. This assay works for most tumours forms and is quick and reasonably priced (Houghton and Taylor, 1977).

#### 2.1.4 Fluorescence

After being treated with drugs, cells are exposed to fluorescently tagged precursors. Using flow cytometry, the fluorescence produced by the replicating cells incorporating the tagged precursor into their DNA is then quantified. Dead or nonreplicating cells are not included in this assay since it measures cells that are actively replicating. The assay is also helpful for adherent and suspension cell lines and does not require radioactivity. Utilising flow cytometry, it is also feasible to ascertain the cells cell cycle phase. Apoptotic cell counts can also be measured. But in order to perform this experiment, the data must be processed using a costly and advanced FACS device. Data on the predictive value for clinical response for this assay is too limited to allow for firm conclusions due to technical challenges in applying flow cytometry to primary tumour tissues (Rotman, 1989).

#### 2.1.5 Dye exclusion assays

Earlier attempts to predict chemosensitivity by excluding essential dyes such as nigrosine, eosin, or trypan blue failed. These tests depended on the cells structural integrity; dead cells would absorb essential dyes like trypan blue since their membranes would no longer be intact. This technique was mainly utilized due to its convenience of handling several specimens and technical simplicity (Isomura et al., 1981). DiSC assay, a new combination of rapid green dye and eosin-haematoxylin, has been utilized more recently by Weisenthal and associates with more encouraging findings, especially in patients with haematologic malignancies such as chronic lymphocytic leukaemia (Weisenthal et al., 1983a; Weisenthal et al., 1983b).

DiSC assay is designed to assess drug-induced cell death in both proliferating and non-proliferating cancer cell populations. It combines rapid green dye with hematoxylin-eosin staining to evaluate cytotoxicity while preserving cellular morphology. In this assay, tumour cells are exposed to test drugs for 4 days, after which dead cells are selectively stained and collected by centrifugation. Internal controls, such as duck erythrocytes, are used to standardize morphological assessment. Although the DiSC assay has shown promising results, particularly in hematologic malignancies like chronic lymphocytic leukemia, no prospective studies have yet demonstrated its ability to reliably predict patient response to therapy (Bosanquet, 1991).

#### 2.1.6 Clonogenic assays

Checkpoint arrest is generally a protective mechanism that allows cells to repair DNA damage, and it does not necessarily trigger cell death. As a result, drugs that induce checkpoint arrest may appear to have a lower IC_50_ value, yet the treated cells may still retain a higher survival rate. In contrast, clonogenic survival assays provide a more direct measure of cytotoxicity by evaluating the long-term ability of a single cell to grow into a colony. This method remains one of the most reliable approaches to assess the true cell-killing potential of anticancer drugs.

Typically, tumour biopsies are processed to obtain single-cell suspensions, which are then exposed to test compounds in order to assess their impact on the colony-forming capacity of cancer cells. A semisolid medium (agar or methyl cellulose) that inhibits the growth of non-malignant cells in the samples is used to rinse and plate the cells. Certain cells will have divided multiple times and developed tumour colonies after 14–28 days; these can be measured visually or semi-automatically. Dead or non-replicating cells are not included in this calculation. The percentage of control growth serves as an indicator of drug activity, and the number of colonies from the treated cells is contrasted with the number of colonies from the untreated control cells. A lengthy incubation period (at least 14 days) before data is provided to the doctor is one of the major technical issues with traditional clonogenic techniques. The test is expensive, time-consuming, and inapplicable to suspension cell lines (Franken et al., 2006).

#### 2.1.7 Cell counting assay

A hemocytometer, or cell counter, is used to estimate the number of cells after they have been cultivated in the presence of the drug for 2 to 5 culture-doubling periods. The assay works with both adherent and suspension cell lines and is quick and simple to execute. However, the cell counter in this experiment can count dead and non-replicating cells. All of the aforementioned tests allow for the calculation of the IC values.

##### 2.1.7.1 Limitations

The cell counting assay is a simple and rapid method suitable for both adherent and suspension cell lines, but it has notable limitations. It cannot reliably distinguish live, dead, or non-replicating cells, potentially overestimating viability. Manual counting is subject to human error, while automated counters may still misidentify cells without additional staining. The assay provides no mechanistic insight into cell death or drug action, and variations in adhesion, aggregation, or detachment during treatment can reduce accuracy and reliability of IC_50_ measurements.

#### 2.1.8 3D tumour models

3D *in vitro* models have revolutionized cancer research in recent years due to its biomimetic property and the ability to accurately depict the *in vivo* situation for drug screening. 3D models are advantageous over the complexity of animal models and the spatial limitations of the cell culture models. In 3D cancer models appropriate matrix components found *in vivo* can be obtained. Cancer cells can be cocultured in a spatially relevant manner with endothelial cells and other cells associated with the *in vivo* scenario. In native tumours, it allows for the monitoring and regulation of oxygen levels to resemble the amounts of angiogenic substances generated by cancer cells in response to hypoxia. For the preclinical assessment of the effectiveness and molecular mechanism of anticancer medications, 3D models offer a promising *in vitro* platform for the aggregation and clustering of cancer cells, migration and proliferation, release of angiogenic factors, and formation of hypoxia within tumour masses (Nyga et al., 2011). An automated assay for 3D models, such as Sphero Chip system has been developed (Kwapiszewska et al., 2014). For long-term 3D cell culture and analysis, this relatively new microfluidic-based device works with commercially available microplate readers. Cultured tumour spheroids grown on a chip can be continuously monitored *in situ* using this device, allowing for the observation of dynamic changes in the cells’ metabolic activity following successive drug dosages. They examined the penetration of synthetic micelles, doxorubicin, and quantum dots into 3D HeLa spheroids versus HeLa cells cultured in a conventional 2D system. A robust and versatile *in vitro* assay was developed to evaluate the penetration dynamics of chemotherapeutic agents and nanomaterials using 3D multicellular tumour spheroids derived from HeLa cells. Unlike traditional 2D monolayer cultures, the 3D spheroid system enabled quantitative assessment of the intratumoural distribution of doxorubicin, quantum dots, and synthetic micelles under microenvironmental conditions that more closely mimic solid tumours. The study demonstrated that HeLa cells grown as 3D spheroids develop key tumour-relevant characteristics including enhanced chemoresistance, altered cellular architecture, and gradients of proliferation and oxygenation that are not recapitulated in 2D culture. This reproducible, image-based, and quantifiable platform therefore provides a more physiologically representative model for preclinical screening and significantly improves the study of drug and nanoparticle penetration into solid tumour tissues (Ma et al., 2012).

3D tumour models offer a more physiologically relevant alternative to 2D cultures but have notable limitations. Their setup and maintenance are complex, requiring specialized techniques, biomaterials, and equipment. Variability in spheroid size, composition, and microenvironment can affect reproducibility and drug response, while these models still lack full *in vivo* interactions such as immune responses and systemic metabolism. High costs, advanced imaging requirements, and computational analysis further limit their scalability. Although promising, 3D models require additional refinement and standardization before routine use in drug discovery.

#### 2.1.9 4D tumour models

A recently developed 4D *ex vivo* lung cancer model generates perfusable tumour nodules on a lung extracellular matrix scaffold, closely recapitulating the histology and protease secretion patterns of human lung tumours. Using the Human One Array v5 microarray platform, gene expression profiles of A549 human lung adenocarcinoma cells cultured in conventional 2D conditions were compared with cells grown within the 4D matrix. Additional comparisons were made with 3D cultures. Gene ontology analysis revealed upregulation of genes associated with extracellular matrix organization, cell polarity, and developmental processes. By supporting larger, perfused tumour nodules, the 4D *ex vivo* model better reflects tumour growth dynamics and microenvironmental interactions observed in patients, providing a physiologically relevant platform for studying lung cancer biology (Mishra et al., 2014).

The *ex vivo* 4D lung cancer model provides a sophisticated platform for replicating tumour microenvironments but has notable limitations. The model is primarily restricted to lung cancer and is costly to maintain and analyze, limiting its suitability for large-scale drug screening. Although it more accurately mimics tumour progression and gene expression, it still lacks systemic factors such as immune interactions, drug metabolism, and distant tissue crosstalk. Additionally, advanced imaging and computational analysis are required, making routine application in early-stage drug discovery difficult.

#### 2.1.10 National cancer institute *in vitro* screening program

NCI-60 is a panel of 60 different human cancer cell lines that covers nine major types of cancer cells. The medicine was tested at five different doses against this varied panel of cell lines, which included lung, colon, CNS, leukaemia, pancreatic, melanoma, prostate, ovarian, breast cancer, and kidney cell lines. It was then incubated for 48 h. Particularly; the screen includes tumours that are resistant to drugs. P-388 murine leukaemia resistant to natural products and human breast cancer selected for MDR are two examples that may help identify new medications with specific efficacy against possibly resistant tumours (Danks et al., 1987; Gupta, 1983). In the screening facilities, up to 200 chemicals can be analysed weekly, or 10,000 annually. In order to test new chemicals at a rate of more than 10,000 per year against a broadly representative panel of 100 or more human tumour cell lines, the NCI has proposed to construct a full-scale screen (Monks et al., 1991). Compounds proceed to the next phase of testing if they exhibit distinctive features, such as selective cytotoxicity toward tumour cell lines, unique mechanisms of action, or efficacy at low concentrations, with only about 2% advancing to mouse studies. Blower et al. analysed microRNA expression profiles across the NCI-60 cancer cell panel and integrated the data into the Cell Miner platform for comprehensive analysis. They found that microRNA-based clustering largely reflected tissue origin, although mRNA expression was slightly more informative across tissues. Notably, microRNA patterns did not strongly correlate with known target transcripts, yet associations between microRNA expression and compound potency suggested a role in chemoresistance. Multivariate analysis integrating gene expression and biological data indicated that microRNA profiles may provide critical insights into the mechanisms governing chemosensitivity and resistance (Blower et al., 2007). A valuable resource for screening drugs against cancer is the NCI-60 cancer cell line panel. However, it has significant limitations. Firstly, the NCI-60 uses pre-existing cell lines that lack adequate representation of the many different types of tumour related factors and the complexity and heterogeneity of real-life tumours (including their microenvironment, immune interactions and vascular components). Also, because the NCI-60 relies on two-dimensional (2D) culture systems, this means that drug response cannot accurately predict what’s happened with *in vivo* tumours or how individual patients may respond to a drug. Second, although it can be useful in understanding how drugs work through resistance mechanisms, the NCI-60 may not necessarily take into account the individual genetic and environmental factors for a given tumour cell. Third, the process of getting samples from test results will take a while and the cost associated with this will limit how many different cancer types could be evaluated and tested.

The NCI-60 panel established high-throughput screening in the 1980s, evaluating 40,000+ compounds annually across 60 human cancer cell lines representing nine tissue types. Five 10-fold dose dilutions generated GI50 (50% growth inhibition), TGI (total growth inhibition), and LC50 (50% lethal) metrics, advancing ∼2% of compounds exhibiting selective cytotoxicity, low nanomolar potency, or novel mechanisms to xenograft testing. Strategic inclusion of resistant models P388 murine leukemia (natural product-resistant) and MCF7 breast (MDR+ multidrug-resistant) enabled identification of clinically actionable resistance mechanisms. NCI-60 throughput reached 200 compounds weekly, screening tumours spanning leukemia, lung, colon, CNS, melanoma, ovarian, renal, prostate, and breast cancers.

Cell Miner suite integrates NCI-60 pharmacology with genomic datasets demonstrated microRNA signatures cluster by tissue origin and predict chemosensitivity (multivariate r = 0.67), with miR-21/155 overexpression conferring doxorubicin resistance across 60 lines. NCI-60 identified >50 FDA-approved drugs including paclitaxel and topotecan (Reinho et al., 2012).

CCLE provides multi-omics for >1,100 lines, correlating KRAS mutations with MEK inhibitor sensitivity (r = 0.82). DepMap’s genome-wide CRISPR screens identify synthetic lethalities MTAP deletion vulnerability to PRMT5 inhibitors validated clinically (85% concordance). CERES algorithm corrects for off-target effects and copy-number biases across 18,881 genes. Pan-cancer CRISPR analyses reveal 760 pan-essential genes and 722 lineage-specific dependencies (Pacini et al., 2021).

Integrated platforms transform precision medicine: DepMap Context Explorer stratifies dependencies by subtype pediatric ALL shows MLLT3 vulnerability absent in adults. CTR2.0 unifies 20 datasets, identifying miR-155 resistance signatures conserved from NCI-60 to DepMap (AUC = 0.84). Recent 7 × 7 matrices across 750+ lines optimize combinations, reducing resistance emergence 60 (Pacini et al., 2024).

CCLE EGFR T790M profiles predict osimertinib response (precision = 87%); DepMap CRISPR data forecasted 82% PARP synthetic lethalities. DepMap/CTR2.0 integration achieves 3.2-fold hit-to-lead improvement, addressing 95% oncology attrition via genomic stratification. NCI-60 for resistance benchmarking; CCLE/DepMap/GDSC for synthetic lethality and subtype-specific discovery (Colombo and Corsello, 2024).

### 2.2 *In vivo* methods

**FIGURE 3:**
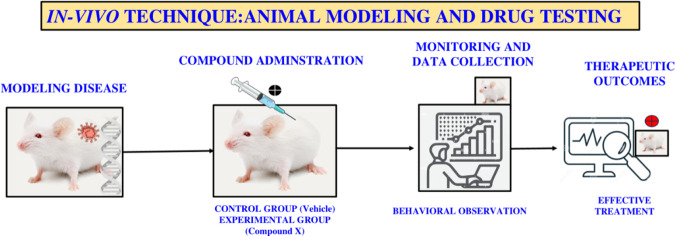
Workflow of *in vivo* techniques for disease modeling and preclinical drug evaluation.

Despite these challenges, animal models remain essential for toxicity evaluation and for assessing the preclinical efficacy of anticancer agents. An important strength of these models is their ability to detect active compounds irrespective of their mechanism of action. Workflow of *In vivo* techniques for disease modeling and preclinical drug evaluation shown in Figure 3. Generally, drugs that show strong and broad activity in animal studies are considered likely to have clinical potential, though exceptions do occur due to species-specific differences in metabolism and cancer biology between humans and rodents. Nevertheless, results from *in vitro* experiments are often reinforced by findings from animal models, underscoring their complementary role in cancer research (Johnson, 1990).

In numerous animal models, the most promising candidate substance is evaluated. The dose-response link, the combined effect of medications, the ways they target cancer, and their organ specificity are all determined. Different dosage forms, dose levels, and animal strains, including animals of specific age groups, can be employed depending on the study design. It is important to select animal models that closely mimic human cancers and demonstrate a meaningful incidence of the disease, ensuring that the results obtained are relevant and translatable to clinical settings (Nuermberger, 2017). *In vivo* anticancer drug screening methods are described under the following headings.

#### 2.2.1 Chemically induced tumour models

Chemically induced models (DMBA, AOM, MNU, OH-BBN) established cancer research foundations but exhibit <10% clinical predictivity. DMBA (7,12-dimethylbenz[a]anthracene) induces skin/mammary papillomas via single topical/IP dosing, modeling PAH metabolism (CYP1A1). AOM (azoxymethane, 30 mg/kg IP) generates colon aberrant crypt foci (ACF) as early biomarkers. OH-BBN (N-butyl-N-(4-hydroxybutyl) nitrosamine, 7.5 mg weekly ×8) produces invasive bladder TCC (40% incidence). These models excel in toxicology screening and mechanistic studies of multistep carcinogenesis (Liu et al., 2015).

However, homogeneous mutations (KRASG12C vs. human polyclonal 10^6^ clones) fail to recapitulate patient heterogeneity. Lacking native stroma, immune interactions, and TME complexity, they generate 70%–80% false positives that fail in PDX validation. Chemical models require 6–12 months versus GEMM/PDX 4–8 weeks, limiting throughput. Modern consensus prioritizes GEMMs/PDXs (65%–85% concordance) over chemical carcinogenesis for therapeutic screening. Historical value persists for regulatory toxicology (ICH S1A) but cannot replace human-relevant platforms (Sewduth and Georgelou, 2024).

Chemical models identified environmental carcinogens reducing human cancer burden 30% since 1970s, yet therapeutic predictivity remains dismal. Current guidelines recommend tiered approaches: chemical models GEMM/PDX, achieving 3-fold hit-to-lead improvement. This rebalancing reflects field shift from historical tools to clinically actionable platforms (Kemp, 2015).

#### 2.2.2 Genetically engineered mouse models (GEMMs)

GEMMs revolutionize cancer modeling with spontaneous progression, native stroma, and immune competence (65%–85% clinical predictivity). KPC pancreatic (KRASG12D; Trp53^+/−^) recapitulates 85% gemcitabine resistance and peritoneal metastasis matching human PDAC. APCmin/+ colorectal models 70% clinical trial correlation via Wnt/β-catenin dysregulation. BRCA1/2; p53−/− breast validates PARP inhibitors (80% sensitivity). Cre-loxP systems enable tissue-specific oncogene/tumor suppressor manipulation, preserving TME fidelity (Pasquali et al., 2025; Kim et al., 2020).

Advantages over chemical models: Predictable latency (8–16 weeks), immune-intact stroma, and multi-hit progression mimicking human genetics. KrasLSL-G12D/+; Ptenfl/fl prostate achieves 92% castration resistance concordance. Unlike xenografts, GEMMs develop spontaneous metastases (liver 40%, lung 25%) via authentic vascular invasion. Single-cell RNA-seq confirms 85% overlap with human tumor transcriptomes (Pasquali et al., 2025).

Species-specific metabolism (CYP3A 10x human), cost ($500K/model), and engineering time (12–18 months). GEMMs excel for immunotherapy (anti-PD1 ORR 35% vs. 100% syngeneic) and stromal-targeted therapies. Combined with CRISPR editing, autochthonous models now test patient-specific mutations in native context. Field standard for mechanism-to-therapy translation, bridging chemical models (10% predictivity) to PDX validation (75%).

#### 2.2.3 Patient-derived xenograft (PDX)

PDXs preserve >90% patient mutations, histology, and stroma through serial passage in NSG mice (75%–85% clinical concordance). Subcutaneous/orthotopic implantation yields 30%–50% engraftment; tumors passage every 2–4 months maintaining heterogeneity. 2,500+ model repositories span breast (85% HER2+ retention), lung (78% EGFR fidelity), and pancreatic cancers. Co-clinical trials demonstrate 12/15 correct PARP inhibitor predictions in ovarian cancer (Janitri et al., 2024).

Superior to cell lines: PDX-derived IC_50_ correlates 0.82 with patient response vs. 0.29 cell lines. Avatar timing enables precision medicine 31% high-risk uveal melanoma patients received PDX-guided therapy post-relapse. Everolimus TGI 55%–78% matched clinical responders. Multi-region sequencing confirms polyclonal architecture absent in engineered models. Murine stroma takeover (P3+), $50K/model/year cost, 3–6-month growth. Humanized PDX (NSG-HSC) predict immunotherapy (70% anti-PD1 accuracy) capturing T-cell exclusion. PDX triaging reduces Phase I failures 60%; NCI Patient-Derived Models Repository validates therapeutic stratification across 30+ indications.

PDX transform drug development 80% negative predictive value prevents futile trials; precision co-clinical trials synchronize mouse/human treatment achieving 85% outcome concordance. Standard for bridging preclinical to clinical translation (Nemati et al., 2023).

#### 2.2.4 Alternative *in vivo* and invertebrate cancer models

##### 2.2.4.1 Zebrafish xenograft models in cancer drug evaluation

Although many compounds show strong anticancer activity *in vitro*, their behavior within a complex multicellular organism can be far less predictable. Rodent models remain the gold standard for evaluating drug toxicity; however, they are not suitable for high-throughput screening of the large number of candidate molecules generated during early drug discovery. To address this limitation, the zebrafish embryo larval system has emerged as a robust, sensitive, and highly predictive model for assessing human toxicity (Garcia et al., 2016). Zebrafish and humans share notable similarities in embryonic development, including conserved gene expression patterns and many comparable anatomical and physiological features. Because therapeutic agents can be introduced directly into the surrounding water, systemic toxicity can be evaluated rapidly. Observable indicators such as reduced viability, morphological defects, and disruptions in development or behavior can be monitored in real time and without invasive procedures, making this model particularly useful for early toxicity profiling (Truong and Tanguay, 2017). In addition to detecting clear morphological abnormalities, a range of specialized tools has been developed to assess drug-induced toxicity in zebrafish. Hematotoxicity is among the most common off-target effects observed with many therapeutic compounds. To evaluate this, a transgenic zebrafish line expressing an erythroid-specific fluorescent reporter has been created, enabling real-time visualization and quantification of changes in blood cell populations following drug exposure. Neurotoxicity can also be assessed using several established behavioral assays, including the widely used photomotor response test, which provides a sensitive measure of drug-induced alterations in neural function (Lenard et al., 2016; Guengerich, 2011; Cassar et al., 2019). The small size of zebrafish embryos and larvae makes them particularly well suited for high-throughput cancer drug screening. During early development, individual embryos or groups of embryos can be accommodated in 96-well plates, enabling simultaneous testing of multiple compounds at a range of concentrations with minimal effort (Hason and Bartůněk, 2019). This format also requires only microliter volumes of media, allowing drug treatments to be performed with very small quantities of test compounds. In addition, the entire organism can be imaged using wide-field objectives, facilitating rapid assessment of treatment-induced phenotypic changes. When coupled with high-content imaging platforms, this approach supports efficient data collection from large numbers of zebrafish xenografts, significantly increasing throughput and analytical power (Leet et al., 2014; Liu et al., 2012).

PDX systems create a very good research model of human cancer. As mice can, zebrafish can provide support for patient donated (tumour) cells/tissues. Use of zebrafish as personalised avatar models to quickly assess the efficacy of therapies is becoming popular (Fazio et al., 2020; Costa et al., 2020). PDX Models of zebrafish have been demonstrated to accurately reproduce a variety of facets of tumour behaviour that occur in patients. For example, breast cancer cells isolated from the bone metastasis of a patient have been shown to migrate to the caudal haematopoietic tissues of zebrafish larvae, which are regions that are similar to human bone marrow, which represents an area in which metastases are generated. Similarly, when intact pancreatic tumour tissue from a patient was implanted into the yolk of embryonic zebrafish, the metastatic behaviour exhibited by that tissue was also consistent with that observed in the patient’s pancreatic tumour (Mercatali et al., 2016).

This system is a promising way to direct individualised therapeutic intervention as well. Biopsy material can be prepared and implanted into zebrafish larvae by established protocols that enable rapid evaluation of drug responsiveness. The advantage of using zebrafish embryos compared to traditional rodent models is that a small amount of tumour cells is necessary for implantation, thereby allowing an increased number of xenografts to be produced from one patient sample and increasing the scope for experimentation while at the same time reducing costs (Corkery et al., 2011).

Having the means to produce many xenografts from one patient sample provides the opportunity to evaluate numerous treatment options at the same time, where the results can be measured in only a few days. We conducted a proof-of-concept study that examined whether it was possible to use zebrafish avatars to test human biopsy samples in zebrafish and determine the effect of bevacizumab (an anti-angiogenic agent) on those samples. This provides a demonstration of how quickly zebrafish-human PDX models can help identify personalized treatment protocols and support clinical decision-making (Rebelo de Almeida et al., 2020).

##### 2.2.4.2 C. elegans

The developmental pattern of *C. elegans*, a minute nematode, has a high degree of predictability. The hermaphrodite form contains a fixed number of 959 somatic cells. All cell division and differentiation events follow a fixed lineage hence, *C. elegans* makes an ideal model to study the disruptive influence of regulatory networks on the balance between cell proliferation, programmed cell death, and differentiation. A limitation for the use of *C. elegans* in these types of studies is that somatic cells in adults no longer undergo cell division, so any investigations on proliferative signaling must be conducted during the larval stages. Another characteristic observed in *C. elegans* is that they are able to display exaggerated cell division but they do not develop true malignant tumours as found in *Drosophila* (Gonzalez, 2013). These nematodes are still a useful functional model organism for studying mechanisms responsible for tumour progression, although *C. elegans* is seen as a flexible and valuable model for many research areas; it relies on the number of conserved human cancer-related genes within nematodes. Mutations in over 1% of human genes have been demonstrated to result in cancers based on the Cancer Gene Census, indicating that a comparison to *C. elegans* counterparts is important (Tate et al., 2019). According to the COSMIC database, about 90 percent of those genes have somatic mutations and about 20 percent contain germline mutations for the cancers included in this analysis. This data demonstrates the contribution both inherited and acquired genetic changes have made toward cancer’s development (Sondka et al., 2018). Approximately half of the genes that carry germline mutations in hereditary cancers also show recurrent somatic mutations in sporadic tumours. To assess the extent to which human cancer driver genes are evolutionarily conserved in *C. elegans*, I used a curated set of 568 driver genes from the IntOGen database, derived from the analysis of more than 28,000 tumours across diverse cancer types. These human driver genes were then compared against the *C. elegans* genome using the Ortholist resource to identify their corresponding orthologs (Martínez-Jiménez et al., 2020; Kim et al., 2018). The analysis revealed that approximately 72% of human cancer driver genes possess one or more identifiable orthologs in *C. elegans*. This proportion is likely an underestimate, as some functional equivalents may be missed due to low sequence conservation. For instance, Ortholist does not classify *cep-1* as an ortholog of the human TP53 gene because of limited sequence similarity. Nevertheless, extensive experimental evidence has established *cep-1* as a true functional ortholog of TP53, demonstrating that sequence-based methods alone may overlook biologically relevant gene relationships (Derry et al., 2001).

Even with the possibility of underestimating the true number of conserved genes, this analysis indicates that *C. elegans* retains orthologs for nearly three-quarters of known human cancer driver genes, underscoring a substantial level of evolutionary conservation. This striking overlap in genetic architecture, together with shared fundamental biological processes, strongly supports the relevance and utility of *C. elegans* as an experimental model for advancing cancer research.

##### 2.2.4.3 Drosophila

Depending on the biological question, cancer models can be generated in either the larval or adult stages of the fly’s life cycle. A variety of human cancers have been successfully modelled in *Drosophila*, including colorectal, lung, thyroid, and brain cancers, among others. In addition, *Drosophila* models are widely used to investigate fundamental molecular processes associated with tumourigenesis, such as the function of tumour suppressors, chromatin regulation, control of cell growth, interactions within the tumour microenvironment, and mechanisms of invasion and metastasis. These features collectively underscore the value of *Drosophila* as a versatile and genetically tractable platform for cancer research (Rudrapatna et al., 2012).

Transgenic *Drosophila* cancer models are most commonly generated using the GAL4/UAS system, which enables controlled spatial and temporal expression of oncogenes or other target genes. By crossing flies that carry tissue-specific GAL4 drivers with those harbouring UAS-linked transgenes, offspring are produced that express the selected gene in defined tissues. The choice of target genes is guided by conserved molecular pathways between *Drosophila* and humans, ensuring biological relevance to the corresponding human cancer type. Expression of these genes triggers cellular and molecular events characteristic of tumourigenesis, which can then be examined in the presence or absence of candidate anti-cancer compounds. Using this system, *Drosophila* cancer models have been instrumental in identifying multiple compounds capable of suppressing aberrant cell proliferation, including several agents already approved by the U.S. FDA. These findings highlight the strong potential of *Drosophila* as a high-throughput and genetically tractable platform for cancer drug discovery and preclinical chemical screening (Munnik et al., 2022).

##### 2.2.4.4 Chick embryo CAM model

CAM model has recently gained substantial attention within tumour biology, imaging, and cytotoxicity research due to its value as an ethical and practical alternative that aligns well with the principles of the 3Rs. This review highlights key concepts that are essential for both new and experienced researchers working with the CAM model, offering foundational information as well as specific methodological considerations relevant to *in vivo* experimentation. In addition, a panel of experts provides insights into some of the most challenging questions in the field through an interview-style discussion (Weber et al., 2021; Miebach et al., 2022; Pion et al., 2022). In the chick embryo, the allantois begins to form around embryonic 3.5 days as an outgrowth from the ventral wall of the endodermal hindgut, projecting into the extraembryonic coelom. As the allantoic vesicle expands rapidly, its mesodermal layer merges with the mesoderm of the neighbouring chorion, giving rise to the CAM (Nagai et al., 2022). This bilayered membrane develops a dense and well-organized vascular network that connects to the embryonic circulatory system through the allantoic arteries and veins. The formation of CAM vasculature occurs in three successive stages, involving both sprouting angiogenesis and intussusceptive microvascular growth (IMG), ultimately producing a robust capillary network that provides an ideal platform for *in vivo* investigations of tumour progression and therapeutic interventions (Schlatter et al., 1997).

To develop a research methodology, the techniques chosen should be those that will minimize animal use, provide reproducible and reliable results, use the species with the least ability to feel pain or distress, and provide data that can safely and accurately be extrapolated to human or other target species. The methodology should also be submitted for review and undergo a regulatory validation process that mandates testing in multiple laboratories to guarantee reproducibility, relevance to *in vivo* outcomes, and review by independent experts before moving on to regulatory acceptance. The chick embryo model satisfies the above requirements and has been shown to be an acceptable alternative to traditional rodent models for use in regulatory toxicology and to provide meaningful data bridging the gap between *in vitro* and *in vivo* studies. The use of the 3R concept will provide for reducing animal use while providing a useful refinement in planning future studies. A careful examination of the results from the application of CAM (chorioallantoic membrane) xenografts will yield valuable preliminary data to assist researchers in determining the effects of their specific gene knockouts or drug treatments on specific cellular functions so that they can use this information to develop hypotheses and design further studies. The CAM model is much more than a preclinical model. It has intrinsic value as a research tool, which will be discussed later in this paper (Vargas et al., 2007).

#### 2.2.5 Engineered and gene-edited cancer models

##### 2.2.5.1 CRISPR-Cas9 engineered lines

The development of experimental animals for accurate *in vivo* cell lineage tracing has traditionally been one of the chief aims of biologists. Traceability studies in mammals have typically relied on dyes and/or fluorescent markers to label and track small populations of cells (Tang et al., 2020). The introduction of CRISPR Cas9 technology has enabled the development of advanced lineage-tracing platforms that exploit the error-prone nature of non-homologous end-joining DNA repair to generate highly diverse, heritable barcodes (McKenna et al., 2016). An initial proof-of-concept study established that CRISPR-based mutation recording could be successfully applied for lineage reconstruction in zebrafish embryos (Alemany et al., 2018; Spanjaard et al., 2018). Subsequent refinements incorporated expressed barcodes, allowing simultaneous assessment of single-cell transcriptomes and lineage relationships. More recently, systems integrating expressed barcodes with transposon-based delivery in the mouse embryo have been reported. These designs rely on constitutive Cas9 activity and multiple genomic target arrays to achieve barcode diversity. Despite their utility, each experiment requires fresh embryonic manipulation, and the resulting animals harbouring numerous randomly integrated transgenes are difficult to maintain as stable breeding lines. Consequently, such models are not well suited for lineage-tracing studies in adult tissues (Chan et al., 2019; Kalho et al., 2018).

##### 2.2.5.2 Cre-LoxP conditional systems

Although previous studies have shown that Cre-mediated recombination efficiency is influenced by multiple variables including the choice of loxP site (wild-type or mutant), the zygosity of the floxed allele, and the age or genetic background of the Cre-driver strain no systematic, large-scale analysis has evaluated the combined impact of these factors at a single, defined genomic locus in mice. To fill this gap, we conducted an extensive multi-locus investigation of Cre recombination across 12 genomic regions distributed on different chromosomes. A major barrier to assessing the influence of inter-loxP spacing has been the lack of reliable methods for precisely inserting loxP-flanked transgenes ranging from 1 to 15 kb. Conventional CRISPR Cas9-based genome engineering remains suboptimal for this purpose because homology-directed repair is inefficient and inconsistent for integrating DNA fragments larger than 1–3 kb (Gurumurthy et al., 2019; Liu et al., 2019). Moreover, accurate placement of loxP sites using CRISPR-Cas9 presents additional technical challenges.

To overcome these limitations, we implemented a high-efficiency Bxb1 integrase system (38–40) to insert 11 constructs-each engineered with distinct inter-loxP distances-into the Rosa26 (R26) safe-harbour locus (Erhardt et al., 2024; Hosur et al., 2022; Low et al., 2022).

### 2.3 Bioinformatics and computational approaches in anticancer drug screening

#### 2.3.1 Bioinformatics in drug discovery

##### 2.3.1.1 Biological database

Bioinformatics is fundamentally based on various types of biological data like genome sequences, proteomic information, gene-expression data, and biomarker information. These biological datasets are produced using numerous techniques and approaches that include molecular biology, cellular biology, and large-scale (high-throughput) experimentation processes. To manage catalogue store, and analyze these biological data using computer-aided tools forms a critical operational part of bioinformatics. Thus, many types of specialized biological databases have been developed to help organize, manage, and share access to these resources. These databases organize not only the original (primary) data but also provide a means of integrating the derived knowledge (e.g., scientific discoveries made through experimentation and technical advancement) into a single database, providing a valuable resource for supporting biological research and expediting the process of drug discovery.

For the last 10 years, bioinformatics using new high-throughput technologies combined with computer-aided design have been an integral part of speeding up modern drug development. These technologies improve the ability to identify, rank and improve upon various natural or man-made compounds and ideally help find the best drug candidates and leading molecules. Additionally, many natural products and their derivatives have been investigated extensively, and these naturally occurring compounds represent about 34% of all newly approved drugs (Patil and Masand, 2021).

One of the greatest global health concerns today continues to be the diagnosis of cancer and the development of targeted therapies has greatly moved us closer to individualized cancer treatment through increasing the quality of care provided to patients. Unfortunately, the complex molecular pathways involved in the development of tumours; the difficulty of developing anticancer treatments; and the need for increased chemical variance and an improved understanding of biological factors require that in depth development efforts can be made on both of these fronts. To accomplish this goal, it is absolutely essential that both libraries of chemicals and datasets of biological information be expandensively developed in order to develop new chemical structures and develop more efficacious anticancer drugs. To meet these needs, numerous specialized databases have been created to systematically curate, manage, and analyze relevant chemical and biological information. Examples include SuperNatural (Dunkel et al., 2006), NPACT (Rosita and Begum, 2020), TCMSP (Ru et al., 2014), CancerHSP (Tao et al., 2015), TCMID (Xue et al., 2013), and Phytochemica (Pathania et al., 2015). These repositories provide structured access to natural compounds, phytochemicals, traditional medicine components, and cancer-related molecular targets, thereby serving as valuable resources for accelerating anticancer drug discovery.

##### 2.3.1.2 Computer-aided molecular docking in drug development

The process of drug discovery is incredibly intricate and challenging, making it all the more important for the selection of the best possible lead compound to determine whether or not a particular project will be successful. The fact that an increasing number of drugs are being developed and entering into human clinical trials at a faster rate than ever before indicates that drugs entering into clinical trials will continue to decrease with regards to their current likelihood of gaining approval from the United States Food and Drug Administration (FDA), thus, providing additional challenges for researchers striving to find more innovative and efficient methods to identify and improve new bioactive compounds. Since the advent of computer aided drug design (CADD), researchers have implemented this strategy as an effective means of guiding experimental studies to recognize and prioritize promising candidate compounds, while reducing the divisions involved in both the cost and duration of conventional methods for discovery. Molecular docking, along with virtual screening (VS) have become integral components of CADD; however; they have also allowed the ability to screen the large population of candidate drugs with the use of more cost-effective methods, by utilizing the resources of high-throughput screening (HTS) more effectively and efficiently (DiMasi et al., 2016).

The evolution of computer technologies and the use of multiple processors have enabled SBDD to develop further; enabling researchers to process target data at an accelerated rate and find strong hit and lead molecules with high accuracy. One of the most widely used methods in SBDD since the late 1980s is molecular docking, a predictive modelling method used to simulate how the ligand binds to its target receptor (binding orientations), identify important interactions, and, in turn, estimate binding affinity; this information will be used during the rational design and optimization of new drugs (Kuntz et al., 1982). Modern drug discovery has multiple applications through molecular docking. It is used to virtually screen chemical libraries, develop leads and optimize them and generate mechanistic hypotheses leading to predictions of mutations. Structural biology research (e.g., X-ray crystallography, cryo-electron microscopy) is aided by molecular docking data when determining the placement of substrates or inhibitors within electron density maps of crystal structures (Stanzione et al., 2021).

Computational screening methods are excellent ways to reduce a large compound database down to a smaller set of compounds that are significantly enriched for compounds considered to have biological activity by producing compounds that are similar to previously identified inhibitors and/or have complementary (potentially binding) characteristics to the target of interest. The most favourable binding orientation, position and conformation of a drug candidate to a protein’s active site can also be predicted through the use of molecular docking, and this information forms the basis for rational lead optimization and provides guidance for future stages of drug development (Ripphaus et al., 2010; Joseph-McCarthy et al., 2007).

##### 2.3.1.3 Omics-driven insights for drug discovery

The drug development and discovery process is an extremely complex and lengthy process that can require a lot of resources to be completed. Drug development and discovery process takes a long time to accomplish and requires a large amount of money to be spent. As productivity declines rapidly, this makes it difficult for the pharmaceutical industry to continue to operate long term. Several factors can impact how effective the drug development pipeline is such as how clearly defined the outcome will be, the expertise level of the medicinal chemists, how good the screening platforms are, and what the total cost of the entire preclinical and clinical development program will be.

Traditional drug discovery methods can be relatively slow, cumbersome, and expensive. Currently, the industry is facing significant challenges due to the lack of a robust late-stage R&D pipeline. In order to overcome these challenges, drug development companies must implement a broad and multi-dimensional approach, which includes rigorous target selection and validation, improvements in preclinical animal models, and the identification of reliable biomarkers and alternatives clinical endpoints. These elements must be addressed in order to improve the efficiency of drug development and to increase the probability of success when developing new drugs.

With the introduction of omics technologies, scientists can now gain a greater understanding of complex diseases and expedite their ability to discover new treatments. The use of multiple omics technologies, such as genomics, transcriptomics, proteomics, and metabolomics, has allowed for an increase in knowledge about the various causes of human disease. The combination of these omics technologies forms an analytical framework that helps to identify how genetic factors contribute to disease through their influence on molecular pathways, cellular processes, and environmental factors. The discovery of the mechanisms of disease across different biological levels will provide valuable assistance in designing and developing effective therapies and in creating precision medicine approaches (Sharma et al., 2023).

##### 2.3.1.4 Genomics in drug discovery

Genes constitute the primary blueprint of existence, defining the entirety of the guidelines which determine biological features, growth, bodily operations and automated cell demise. The data stored in the human genome has become a necessity in the current drug research and development. Data derived through genomes aid in the identification and validation of therapeutic targets, direct the evaluation of target-compound interactions, and aid in identifying clinically meaningful trial endpoints. In 2003, the Human Genome Project was finished, and that event was a significant breakthrough in the biomedical field, triggering the fast growth of the so-called omics technologies. The further developments in high-throughput sequencing have made it possible to identify genes and their functions on a large scale and the further decrease in the sequencing costs has opened up the genomic research activities to a very large number of organisms other than human beings. This growth has already made comparative genomics an effective means of identifying pathogen-specific weaknesses and guiding the development of specific therapies. The further democratisation of genomic technologies among researchers has also been achieved by increased accessibility to sequencing platforms.

**FIGURE 4:**
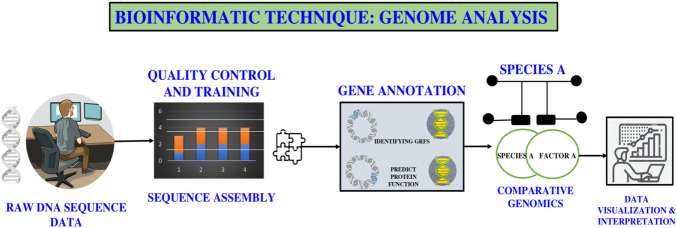
Genome analysis workflow in bioinformatics.

These inventions have strengthened this interpretation that proteins coded by the genome are the main functional outcome in biological systems and form the largest fraction of viable targets of the drug. By closely analysing the DNA sequences and understanding of genetic variation, genomics offers the accuracy required to determine the disease-related mutations and rank the candidate targets to therapeutic intervention. Description of the genome analysis pipeline, including the main stages of processing of raw DNA data, annotation of genes, comparative genomics, and ultimate interpretation of data as presented in Figure 4. With the maturation of these technologies, there will be a vast proliferation of genomic data being incorporated into the various platforms and analysis frameworks, which will greatly accelerate the overall drug discovery and development pipeline (Finan et al., 2017).

### 2.4 Advanced translational preclinical cancer models

#### 2.4.1 Patient-derived organoids (PDOs)

Patient-derived organoids (PDOs) are 3D, stem cell-based mini-organs cultured from patient tumour biopsies that self-organize to recapitulate native tissue architecture, genetic heterogeneity, and functional complexity. Unlike 2D cell lines, PDOs preserve epithelial-stromal interactions, crypt-villus structures (intestine), ductal organization (pancreas), and intratumour heterogeneity key drivers of therapeutic resistance missed by traditional models. Generated via enzymatic dissociation and embedding in Matrigel with Wnt/R-spondin/Noggin growth factors, PDOs expand >100-fold within 4–6 weeks while maintaining patient-specific mutations (TP53, KRAS, APC) (Acar, 2025).

PDO biobanks now span colorectal (90% success), breast (70%), pancreatic (60%), and lung cancers, enabling personalized drug screening. Positive predictive value reaches 85%–93% for chemotherapy response in colorectal/prostate cancers, far surpassing cell lines (r<0.3). PDOs predict irinotecan resistance via SLFN11 expression and guide PARP inhibitor selection in ovarian cancer. Limitations include absent vasculature/immune cells and culture failure rates (10%–40%). Despite this, PDOs reduce the 95% attrition rate by identifying responders pre-trial, with 80% concordance to patient avatars (Wensink et al., 2021).

#### 2.4.2 Patient-derived xenografts (PDXs)

Patient-derived xenografts (PDXs) involve implanting fresh human tumour fragments directly into immunocompromised mice (NSG/NOD-SCID), preserving original histology, genomics, and stroma through serial passage. Unlike cell line xenografts, PDXs maintain >90% of patient mutations, copy number variations, and tumour microenvironment for 5–10 passages. Subcutaneous, orthotopic (liver, pancreas), or metastatic site implantation yields 30%–50% engraftment success, with tumours passaged every 2–4 months (Xu et al., 2019).

#### 2.4.3 Humanized mouse models

Humanized mice are immunodeficient strains engrafted with human CD34^+^ hematopoietic stem cells (HSCs) or PBMCs, reconstituting a functional human immune system alongside PDX/PDOs for immunotherapy testing. NSG-SGM3 mice achieve 25%–50% human chimerism with mature T/B/NK cells, monocytes, and dendritic cells within 12–16 weeks post-HSC transplant. “Onco-Hu” variants combine PDX tumours with immune reconstitution, modeling checkpoint blockade and CAR-T responses.

These models predict clinical immunotherapy outcomes with 70%–85% accuracy, capturing T-cell exhaustion, cytokine release syndrome, and MHC-restricted responses absent in standard xenografts. Anti-PD1 response in humanized NSCLC PDX (30% ORR) matches KEYNOTE-001 trial data. Graft-versus-host disease (20% by week 20), incomplete lymphoid architecture, and poor memory B-cell function. Despite constraints, humanized mice validate bispecific antibodies and identify MHC-I loss as resistance mechanisms, bridging the 95% translational gap (Walsh et al., 2017).

#### 2.4.4 Microfluidic/organ-on-chip systems

Organ-on-chip (OoC) devices are microfabricated PDMS chips with continuously perfused chambers containing human cells arranged to mimic tissue interfaces, vascular flow, and physicochemical gradients. Lung-on-chip recapitulates alveolar-capillary barriers with cyclic stretch; gut-on-chip models peristalsis and microbial crosstalk. Multi-OoC “body-on-chip” platforms interconnect liver, tumour, and bone marrow chips via vascular channels, replicating whole-body pharmacokinetics (Bhatia and Ingber, 2014).

### 2.5 Biological complexity of the tumour microenvironment

#### 2.5.1 Tumour microenvironment (TME) complexity

The tumour microenvironment (TME) comprising immune cells, cancer-associated fibroblasts (CAFs), extracellular matrix (ECM), blood vessels, and signaling gradients dominates therapeutic response more than tumour genetics alone. TME generates hypoxia (pO_2_<10 mmHg), acidosis (pH 6.5–6.8), and nutrient deprivation that induce resistance via HIF1α, MDR1 upregulation, and autophagy. ECM stiffness (10–100× normal) activates YAP/TAZ signaling, promoting invasion and stemness absent in 2D cultures. Traditional *in vitro* assays (MTT, clonogenic) ignore these biophysical barriers, generating 70%–80% false positives that fail *in vivo* (Wu and Swartz, 2014).

Vascular abnormalities leakiness (300 nm pores), high interstitial pressure (40–60 mmHg), and hypoxia restrict monoclonal antibody penetration to tumour rims (<100 μm). Organoids/PDOs capture epithelial organization but lack functional vasculature and immune infiltration, missing perfusion-dependent drug delivery failures. PDXs preserve human stroma initially (P0-P2) but murine fibroblasts dominate by P5, altering cytokine profiles. Microfluidic tumour-on-chip models recapitulate 90% of these gradients but remain low-throughput. TME-mediated resistance explains why 95% of preclinical hits fail clinically nanoparticles aggregate in necrotic cores, chemotherapeutics degrades in acidic ECM, and immunotherapies encounter exclusion zones (Crouigneau et al., 2024).

TME “reprogramming” creates self-sustaining loops: tumour-derived VEGF recruits endothelial cells while CAF-derived TGF-β activates myCAFs, compressing vessels and exacerbating hypoxia. This dynamic reciprocity selects resistant clones, underscoring why static 2D models predict <20% of patient responses while multi-cellular 3D platforms achieve 60%–85% concordance. Addressing TME complexity requires hybrid platforms integrating flow, stroma, and immunity (Baghban et al., 2020).

#### 2.5.2 Immune interactions and evasion

Immunosuppressive TME enables >90% of clinical immune evasion through exclusion (no T-cell infiltration), dysfunction (exhausted PD1+ TIM3+ T-cells), and active suppression (Tregs, MDSCs). Preclinical models lacking human immunity systematically overpredict immunotherapy efficacy standard xenografts/PDXs have no lymphocytes, yielding 100% false “response” rates to checkpoint inhibitors. Cell lines ignore MHC-I downregulation and antigen loss variants that comprise 40%–60% of resistance mechanisms (Mai et al., 2024; Yamaguchi and Perkins, 2018).

Key evasion pathways absent in traditional screening: TGF-β creates “cold” exclusion zones (85% of PDAC); adenosine (CD73+CAFs) paralyzes T-cell signaling; IDO1 catabolizes tryptophan, starving effector T-cells. Organoids/PDOs exclude immune co-culture (success <30%), missing TAM-mediated VEGF production that blocks 70% of anti-PD1 responses. Humanized mice (NSG-HSC) achieve 25%–50% chimerism but graft-versus-host disease limits duration to 16 weeks. PDX “avatars” predict 75% of pembrolizumab responses but murine stroma dilutes immune fidelity (De Cicco et al., 2020; Tufail et al., 2025).

Tumour-immune crosstalk creates feedback loops absent in monotherapy screening: IFN-γ inducible PD-L1 expression, VEGF-mediated Treg recruitment, IL-10 suppression of DC maturation. Microenvironment gradients (hypoxia→HIF1α→ARG1+ MDSCs) create niches where 60%–80% of tumour mass evades adaptive immunity. Clinical trials confirm: tumours with >20% tertiary lymphoid structures respond 4× better to immunotherapy. Current models capture <15% of these dynamics, explaining 85% immunotherapy attrition. Multi-cellular platforms with human NK/T-cell co-cultures and cytokine gradients achieve 70%–85% prediction accuracy (Haist et al., 2021).

#### 2.5.3 Stromal heterogeneity and cancer-associated fibroblasts (CAFs)

CAFs comprise 30%–70% of tumour mass, exhibiting greater transcriptional diversity than cancer cells across single-cell RNA-seq of 100+ patients. Two dominant subtypes myCAF (ACTA2+αSMA+, matrix-producing) and iCAF (IL6+STAT3+, inflammatory) create distinct resistance niches. myCAFs generate collagen VI/IX barriers (100 μm thick) that exclude therapeutics; iCAFs secrete HGF, FGF, and IL6 activating PI3K/mTOR in 80% of resistant clones. Cell lines eliminate this heterogeneity entirely (Vickman et al., 2020).

CAF-tumour co-evolution drives 85% of therapeutic resistance through epigenetic plasticity: TGF-β1 induces myCAF trans differentiation (85% FAP+); cancer-derived IL6 sustains iCAF via STAT3 (70% tumours). Single-cell analysis reveals 12 CAF clusters with distinct ECM gene signatures LOXL2+ subtypes correlate with 3x worse survival. PDXs retain human CAFs for 2 passages then murine takeover; organoids exclude stroma completely. Organ-on-chip with primary CAFs restores 90% of resistance patterns seen clinically (Wang et al., 2026).

Stromal metabolic coupling sustains cancer under therapy: CAFs export lactate fueling OXPHOS in cancer cells (Warburg reversal); glutamine from CAFs activates mTORC1. CAF-derived exosomes transfer miR-21, enhancing chemoresistance via PTEN suppression. Spatial transcriptomics shows CAF-cancer distance <50 μm predicts recurrence. Heterogeneity explains why VEGF inhibitors fail (60%): CAFs produce FGF2/PDGF, activating alternate angiogenesis pathways. Anti-CAF therapies (TGF-βR inhibitors) show 65% synergy with chemotherapy in trials. No current model fully recapitulates 10+ CAF subtypes driving clinical failure (Patel and Singh, 2020).

## 3 Other models

Apart from animal models used at NCI, a few other important models of chemical carcinogenesis are as follows.

### 3.1 DMBA-induced oral cancer in hamster

Male Syrian hamsters can be experimentally induced to develop oral squamous cell carcinoma by topical application of 0.5% DMBA dissolved in liquid paraffin. Approximately 10 µL of this solution, equivalent to 100 µg of DMBA, is carefully applied to the right buccal pouch mucosa three times per week for 16 consecutive weeks. Throughout the experimental period, animals are monitored for clinical signs of tumour development. At the end of the treatment period, the tumours are evaluated for number, size, and total tumour burden. Tumour size can be measured using digital calipers or imaging software to calculate tumour volume, and histopathological analysis may be performed to confirm malignancy. These measurements are then compared with those from the control group, which receives either no treatment or vehicle-only applications, to assess the carcinogenic effect of DMBA and the efficacy of any experimental interventions (Balasubramanian et al., 1995).

### 3.2 DMBA sustained release suture technique

Squamous cell carcinoma can be induced in the cheek pouch of Syrian hamsters using a sustained-release DMBA suture in combination with the tumour promoter arecaidine. Tumours reached an average size of 100 mm^2^ in 80.6% of treated animals. Treatment-related adverse effects included mortality in 15.8% of animals and severe local inflammation in 3.6%. Tumour development was monitored through regular clinical assessments, and tumour size was measured using standardized caliper methods. This model provides a reliable platform for studying squamous cell carcinomas of the upper aerodigestive tract, enabling investigations into tumour biology, progression, and therapeutic interventions (Wani et al., 2001).

### 3.4 3-Methylcholanthrene-induced fibrosarcoma tumours in mouse

MCA is a potent chemical carcinogen used to induce subcutaneous fibrosarcomas in mice. In this model, MCA 200 µg of 100 µL DMSO is injected into the thigh area, single subcutaneously. The development of the tumours normally starts 6–7 weeks after the injection and the tumour incidence is about 90% after 15 weeks. During the experimental time, the tumour growth (volume and latency) is observed in the experimental groups and the control groups and compared. Assessment is done every week to record the developmental stages and progress of fibrosarcomas. Focal subcutaneous necrosis and fibrosarcomas are always observed in the histopathology of MCA-injected mice, which proves the high tumorigenic potential of MCA. This model presents a stable base to investigate the biology, disease progression and efficacy of anticancer therapeutics of fibrosarcoma (Elangovan et al., 1994).

### 3.5 3-Methylcholanthrene-induced skin tumours in mouse

Experimentally, skin tumours in mice are induced by topical application of 0.05 mL of 0.30 M CA solution on the shaved back of the body twice a week. The treatments are carried on until macroscopically visible tumours form. During the 15 weeks of experimental work, the incidence, multiplicity, and latency of tumour are periodically recorded and checked in comparison to the treatment of MCA and the control. The evaluation of tumours involves visual examination, determination of the size of the tumour through calipers and confirmation through histopathological analysis in cases of doubt. With this model, tumour incidence has been reported to be up to about 85 and this shows the strong tumorigenic capacity of MCA to induce cutaneous neoplasm. This system represents a stable platform on which the study of skin carcinogenesis and the effectiveness of the possible chemopreventive or therapeutic agents could be studied (Ohkoshi and Fujii, 1983).

### 3.6 Benzopyrene-induced forestomach tumours in mouse

A good example of a potent environmental carcinogen and representative polycyclic aromatic hydrocarbon is benzopyrene that is able to induce tumour development in rodents. In this model benzopyrene is orally administered to mice at a dose of 1 mg in 1.1 mL of peanut oil after every 4 weeks in the form of a two times, 5-min oral gavage. The regimen causes the growth of forestomach tumours. During the experimental time, the animals are followed up in terms of clinical indicators, and the rate of tumour occurrence, multiplicity and load are determined and compared with the untreated control mice.

Tumour identification is confirmed through gross examination and, when necessary, histopathological analysis. Historical and experimental observations indicate that benzopyrene treatment can result in up to 100% incidence of forestomach tumours, demonstrating the high carcinogenic potential of this compound. This model provides a reliable platform for investigating the mechanisms of chemically induced forestomach carcinogenesis and evaluating potential chemopreventive or therapeutic interventions (Azuine and Bhide, 1992).

### 3.7 Hepatocellular carcinoma

HCC can be experimentally induced through different chemical agents. Several well-established animal models are available for studying HCC, including naturally occurring models such as woodchucks infected with woodchuck hepatitis virus and Long-Evans cinnamon rats, which develop liver disease due to abnormal copper accumulation similar to Wilson’s disease. In addition, chemically induced models are also widely used to replicate the process of liver carcinogenesis (Tennant, 2001; Yoon et al., 2000; V and esslinovitch, 1990). Among available animal models for HCC, woodchucks chronically infected with woodchuck hepatitis virus provide a highly relevant system for studying liver carcinogenesis; however, their maintenance is technically challenging and resource-intensive. Alternatively, HCC and associated liver lesions can be experimentally induced in male B6C3F1 mice through a single intraperitoneal injection of ENU at a dose of 120 μg/kg. Animals are monitored longitudinally and sacrificed 60 weeks post-exposure for evaluation. Tumour development is assessed through gross examination of the liver, measurement of tumour size and number, and histopathological confirmation. This chemically induced mouse model offers a practical and reproducible platform for investigating HCC pathogenesis and testing potential therapeutic interventions (Di Bisceglie et al., 2005). Chemically induced rodent models of HCC offer several advantages for preclinical research. These models are relatively easy to maintain and provide reproducible experimental outcomes. Their high incidence of HCC makes them valuable systems for evaluating potential chemopreventive or therapeutic agents. The duration of the study, average of 60 weeks, is quite similar to the chronic course of HCC in man, which normally takes years of persistent necroinflammation and fibrosis as a result of viral or non-viral liver damage. Also, the estrogen receptors have been shown to be present in chemically induced HCCs in rodents and this indicates that they are also useful to study the hormone-related mechanisms of liver carcinogenesis (Alagaratnam et al., 1987).

### 3.8 Hepatocarcinogenesis in MDR2 knockout mice

MDR2-KO mice, which do not have the canalicular phosphatidylcholine transport protein, P-glycoprotein are used as a model of inflammation-linked HCC. Lack of MDR2 causes the deficiency of phospholipid and causes reflux of bile and long-term inflammation in the portal. Sustained inflammatory injury promotes hepatocyte dysplasia and the subsequent development of HCC, closely recapitulating the sequence of liver disease progression observed in humans (Smit et al., 1993; Fickert et al., 2004; Mauad et al., 1994). In MDR2-KO mice, the liver at 3 months of age demonstrates increased hepatocyte DNA synthesis and upregulation of antioxidant defense mechanisms. Expression of cell proliferation markers, including Cyclin D1 and PCNA, is significantly elevated; however, hepatocyte mitotic activity remains suppressed at this stage. During the later stages of disease progression, hepatocyte mitotic activity increases despite a reduction in inflammatory responses and normalization of overall hepatic antioxidant capacity. These findings indicate a temporal dissociation between proliferative signaling, mitotic activity, and oxidative stress during the pathogenesis of liver disease in MDR2-KO mice (Katzenellenbogen et al., 2006).

### 3.9 High fat diet induced NAFLD/NASH model in mouse

Male C57BL/6J mice fed a high-fat diet for 60 weeks develop obesity and insulin resistance, resulting in NASH and HCC. This model recapitulates the typical progression of NAFLD to NASH, including hepatic steatosis, pro-inflammatory cytokine release, oxidative stress, hepatocyte injury, and fibrosis, ultimately leading to carcinogenesis. Nevertheless, some features of the model do not match human NASH, such as metabolic responses of species and faster progression of the disease, which must be taken into account when one interprets the results of the experiment (Nakamura and Terauchi, 2013).

### 3.10 Pancreatic cancer models

The exocrine pancreatic cancer research has also been considerably enhanced by GEMs since it has played a role in the study of pancreatic dysplasia, neoplasia and chronic pancreatitis in a physiologically relevant *in vivo* setting. Such models are most commonly defined based on a keen histological and immunohistochemical studies to determine the tumour initiation, progression, and molecular changes. Although holding promise, the capability of GEMs to forecast therapeutic outcomes in patients with pancreatic cancer is questionable when it is compared to recommended FDA xenograft models. In order to optimize translational relevance, experiments using GEMs must be developed with a stringent experimental design such as standardized protocols and thorough phenotypic characterization which is similar to the infrastructure used to conduct properly done clinical trials (Hruban et al., 2006).

### 3.11 Angiogenesis assays

These assays often utilize transplantable tumour models, enabling the assessment of both mechanistic and functional responses to candidate compounds. Several established cell lines have been identified as suitable for inoculation in these models, providing reproducible systems for studying angiogenesis and therapeutic efficacy (Phung and Dass, 2006).

## 4 Methods involving cell line/tumours pieces implantation

### 4.1 Transplantable tumour models

Transplantable tumour models offer a rapid and reproducible system for evaluating anticancer agents, as tumours develop more quickly than in chemically induced models when a defined number of cells from a given cell line are implanted into a susceptible mouse strain. Commonly employed cell lines include P-388 and L-1210, derived from murine lymphocytic leukemia, which exhibit near-complete proliferative capacity. The tumour burden and host survival can be precisely predicted based on the number of cells inoculated, allowing assessment of therapeutic efficacy by measuring tumour growth inhibition and extension of animal survival.

Other frequently used transplantable tumour cell lines include B-16 melanoma, Lewis lung carcinoma, and sarcoma-180. Appropriate host strains vary: BDF mice are typically used for most cell lines, while Swiss mice are used for sarcoma-180. Inoculation routes are selected based on tumour type, including intramuscular injection for Lewis lung carcinoma, subcutaneous injection for sarcoma-180, both intraperitoneal and subcutaneous injection for B-16, and intravenous or intraperitoneal injection for P-388 and L-1210. These experiments are generally completed within approximately 10 days, providing a rapid and reliable platform for screening candidate anticancer compounds (Guerin et al., 2020).

Mean survival time (MST) is calculated as:

$$\frac{T}{C}\%=\left(MST\;of\;the\;treated\;animalsT\right)/\;MST\;of\;the\;control\;animals\left(C\right)\times 100$$

For sarcoma 180 tumours, reduction of tumour size (tumour weight) is used to find out the tumour inhibiting activity of solid tumours as follows:

$$\text{Tumour inhibiting activity }\left(\text{TIA}\right)=\frac{Average\;tumour\;weight\;of\;the\;treated\;animals\;\left(T\right)}{Average\;tumour\;weight\;of\;the\;control\;animals\;\left(C\right)}\times 100$$

### 4.2 Hollow-fiber technique

In this study, each mouse received six polyvinylidene fluoride (PVDF) hollow fibers containing three gastric or hepatic cancer cell lines, with three fibers implanted subcutaneously and three intraperitoneally per mouse. Six mice were used in total, divided equally into paclitaxel-treated and vehicle control groups. PVDF fibers (1.0 mm inner diameter, 500 kDa molecular weight cutoff) were conditioned by soaking in 70% ethanol at room temperature for 72 h, followed by thorough rinsing with deionized water, sterilization via autoclaving, and preconditioning in RPMI-1640 medium supplemented with 20% FBS. Cancer cells were harvested using trypsin-EDTA, centrifuged, and subsequently resuspended in conditioned medium at an optimal density to maintain continuous linear growth throughout the study period. A 20-gauge needle was used to fill the fibers with the cell suspensions and preheated clamps were used to heat-seal the fibers at intervals of 2 cm. Before implantation, fibers were cultured overnight or one to three nights according to the rate of proliferation of a specific cell line at 37 °C in a humidified 5% CO_2_ environment. The model is a hollow fiber PVDF with the ability to evaluate tumour cell response of therapeutics *in vivo* by keeping microenvironment conditions controlled in the fibres, which can precisely determine drug efficacy.

Implantation was performed on day 0 by implanting female Balb/C (nu/nu) mice aged 7–10 weeks under the influence of isoflurane anaesthesia. In case subcutaneous implantation, 3 PVDF fibers were laid at the back of the neck with the help of a trocar. In the case of intraperitoneal implantation, other three fibers were placed through a small abdominal incision and fixed to the peritoneal and abdominal wall using sutures. The viability of the tumour cells in terms of mass was evaluated after 5 days in the fibers. Duplicate *in vitro* control fibers were cultured to give baseline comparisons.

The treatment process of the drug therapy was initiated three to 4 days after the implantation, and thereafter was given every day and provided on a daily basis over a period of 4 days. One day following the last dose, mice were sacrificed and the viable cell content in each fiber of the mice was measured by taking the MTT assay. Antitumour activity was evaluated based on the comparison of the viable cells mass between drug treated group and control groups. To confirm the experimental model, two compounds, EW7197 and flavopiridol, were experimented using SNU-16 human gastric cancer cells, but using orally and intraperitoneally administered, respectively. This methodology can be used to accurately assess tumour cell response *in vivo* in a controlled microenvironment, allowing drug efficacy to be assessed and reproducible and quantifiable endpoints to be measured (Hollingshead et al., 1995).

### 4.3 Use of xenografts

In this xenograft model, human tumour cells are subcutaneously implanted into immunodeficient mice to evaluate the *in vivo* potency of potential anticancer drugs. Those compounds that show potency in initial screens, such as hollow fiber systems, are given in a dose-response manner, and only those that can inhibit or regress tumour growth with minimal systemic toxicity are selected for further preclinical studies. The assay takes about 30 days. For tumour establishment, 1 × 10^7^ cells from each human cancer cell line, resuspended in 100 µL serum-free RPMI medium, were mixed with an equal volume of Matrigel (BD Biosciences, Bedford, MA) and injected into the subcutaneous flank of NOD/SCID mice. After the establishment of tumours, they were removed, cut into ∼3 mm fragments, and passage into the flanks of additional NOD/SCID mice for large-scale evaluation of drug response. All experiments were conducted with tumours at the third *in vivo* passage for SNU-16, SNU-668, SK-Hep-1, HepG2, and Hep3B cell lines, and at the fourth passage for SNU-484. Tumour volume (V) was calculated using formula V = ½ × a × b^2^, where a is the longest diameter of the tumour and b is the shortest, both measured in millimeters. When tumours reached approximately 100 mm^3^, mice with comparable tumour sizes were randomly assigned to experimental groups using SAS software. Each treatment and control group consisted of three animals. Treatment commenced immediately after group allocation, with either the test compound or vehicle control administered for five consecutive days at the same dose and via the same route as in the hollow fiber assay.

Antitumour efficacy was evaluated using the percentage treated/control (%T/C), defined as the ratio of the median tumour volume in the treated group (T) to that of the control group (C) on the day of measurement. In instances of tumour regression, %T/C was expressed as the negative percentage change in the median tumour volume of the treated group relative to baseline. The lowest %T/C value observed within the first 7 days of therapy was considered the optimal indicator of xenograft responsiveness for each drug within the respective tumour model (Lee and Rhee, 2005).

### 4.4 Spheroid culture of LuCaP 147-induced prostate cancer

A prostate cancer model was established by serially passaging spheroid cultures derived from LuCaP 003147 xenografts. This approach demonstrated the utility of spheroid systems for high-throughput drug screening and for evaluating the effects of anticancer agents on cell cycle progression and apoptosis. Notably, cells derived from these spheroids were capable of forming tumours upon transplantation back into mice, providing a robust *in vitro* and *in vivo* preclinical platform. This model represents a specific subtype of prostate cancer characterized by a hypermutator phenotype and the presence of an SPOP mutation, making it particularly relevant for studies of molecularly targeted therapies (Saar et al., 2014).

### 4.5 Integration free-induced pluripotent stem cells (iPSCs) model

In this experimental platform, isogenic human ESC lines deficient in FANCA were generated to model the genetic and cellular defects associated with FA. iPSCs were obtained out of a patient with no genetic complementation, which offers a genetically relevant and patient-specific cellular system. *In situ* gene repair was also effectively executed in these FA-iPSCs, and FANCA came to be expressed functionally, and these findings showed that accurate genome editing could be successfully done in a clinically applicable situation. The system, in addition to re-epitomizing the hematopoietic defects that are typical of FA, offers a flexible platform that can be used to conduct high-throughput screening of drugs. It will be possible to identify candidate compounds that increase the proliferation, survival, and differentiation of hematopoietic progenitors of FA-iPSCs providing insights into possible therapeutic interventions. This method will enable investigation of the pathophysiology of diseases, studies of genetic rescue therapy, and testing of pharmacological interventions in a human-specific and controlled environment by patient-derived iPSC combined with target gene correction. This model is, therefore, a strong preclinical instrument in the development of precision medicine and also regenerative treatment of Fanconi anemia (Liu et al., 2014).

## 5 Nude mouse model

The use of nude mice dates back to cancer research especially to measure the tumorigenic properties of cells and the effectiveness of anticancer drugs. The characteristic features of these animals are the congenital lack of thymus that makes them immunologically defective. Therefore, they cannot execute a mitotic reaction during mixed lymphocyte reactions and cannot produce cytotoxic effector cells. The depletion of both the helper T cells and suppressor T cells also results in alteration in the production of the antibodies in the presence of the antigens. Moreover, nude mice lack contact sensitivity and can neither reject transplanted tissue nor cells and hence they can be used well in xenograft studies. But their maintenance needs high standards of sterile housing and strong controlled conditions of hot temperatures (26–28 °C) to avoid infections and survival.

Some types of tumours show high proliferation rates in these mice, e.g., melanomas and colon carcinoma, and others, e.g., prostate carcinoma and most leukaemia show poor proliferation. Avoiding necrosis in a tumour requires inoculation with a large number of cells, typically of the order of 10^6^, under the skin. Although the induction of tumours has been successful, metastasis is seldom witnessed in nude mice; this is a weakness of this model. Moreover, the general care of these animals is expensive because of the specialized care and sterile conditions as well as facilities. However, their distinctive immunodeficient condition remains to render them an invaluable example in the study of cancer (Szadvári et al., 2016).

## 6 Newborn rat model

It is urgently required that new treatment modalities of paediatric brainstem tumours be developed because the blood-brain barrier does not allow most treatment modalities to access these malignant cells. In order to evaluate the feasibility of direct, localized drug delivery, scientists created experimental brainstem tumour models in adult rodents (Wu et al., 2002). Neonatal tumour models are either produced through the exposure of pregnant animals to carcinogenic agents or inoculation of the newborn animals with tumour cells. However, carcinogen-treated expectant animals are not as appropriate in assessing the efficacy of drugs because of a number of limitations that are inherent (Peterson et al., 1994). The agents introduced as carcinogenic agents during pregnancy tend to be unevenly distributed resulting into random tumour development. Consequently, not all the offspring are affected and others may have tumours which are heterogenous and of various sizes with some being small lesions and some large tumours. Direct inoculation, by comparison, offers the advantage of inoculation of more controlled and uniform tumour burden. Nevertheless, it is yet unknown whether the developing brainstem can withstand the inoculation procedure as well as the subsequent implantation of tumour cells. To compound the situation, the brainstem lacks the same level of functional redundancy as the cerebral cortex, i.e., even trivial surgical manipulations of this area may lead to a considerable threat of the impairment of neurological functions and morbidity (Klopp et al., 2000). The neonatal tumour models are more accurate representations of paediatric cancers and can be used in the development of the translational research. Instead of using nude mice, newborn rat pups are frequently utilized as the alternative option in terms of tumour transplantation because they are cheaper, easier to maintain and can also be reliably propagated to produce transplantable tumour cell lines. These models are especially useful for investigating neural tumours. For example, under sterile conditions, 1 × 10^6^ viable C6 glioma cells suspended in 10 µL of phosphate-buffered saline can be injected into the left hemisphere of rat pups. Following inoculation, the animals are observed twice per week to monitor tumour progression. In Sprague-Dawley rats, palpable C6 glioma xenografts usually develop within 15–20 days after transplantation (Jallo et al., 2005).

## 7 Transgenic mouse model

Cancer is fundamentally a genetic disease, with many human malignancies arising from mutations in oncogenes or tumour suppressor genes. Targeted inactivation of specific genes within particular tissues of adult mice provides a powerful model to replicate the somatic mutations observed in human cancers. Genetically engineered mice not only serve as valuable systems to study disease mechanisms but also offer platforms for evaluating potential gene therapy strategies. Such models can be created either through pronuclear DNA injection or by gene-targeting techniques (Lovejoy et al., 1997). The Meta Mouse is a genetically engineered animal model that has recently been granted a U.S. patent. Unlike conventional xenograft models where a suspension of tumour cells derived from established cell lines is injected beneath the skin of nude mice the Meta Mouse involves transplanting actual tumour fragments from patients directly into the corresponding organ of origin. This approach allows the tumours to grow in a more natural microenvironment. As a result, the Meta Mouse develops disease features such as metastasis and cancer-related weight loss in a manner that closely resembles human pathology. By contrast, traditional mouse models rarely show metastasis, as the process is either absent or progresses too slowly. This limitation is one of the major reasons why many drugs that appear promising in preclinical xenograft studies ultimately fail in clinical trials.

Tumour progression has been suggested to be particularly dependent on the cell-to-cell interactions (Tetsuro Kubota). Under traditional conditions, whereby the tumour cells are dissociated into suspension, the application of enzymes to degrade tissues may destroy important surface receptors required in such interactions hence changing the tumour behaviour. The Meta Mouse overcomes this limitation and helps to develop various forms of cancer in humans such as liver, pancreatic and head and neck cancer, bladder, stomach, ovary and colon cancer, lymphoma. It is also the sole model of mesothelioma that is in existence now. Nevertheless, in this model, breast and prostate cancers have a slower rate of metastasis. The Meta Mouse finds extensive application especially in the assessment of new drug formulations, routes of administration and dosing approaches and even new uses of existing drugs. Importantly, it can act as a surrogate marker for patient response, enabling clinicians to better predict prognosis and personalize treatment choices by testing therapies on the patient’s own tumour grown in the model. Despite its advantages, the major drawback of this system is the long duration required for studies, which can limit its practicality in fast-moving clinical settings (Holzman, 1996).

## 8 Organoid cancer modeling

The development of organoids as an *ex vivo* model system has revolutionised primary and clinical cancer research over the past 10 years. Organoids are tiny reproductions of human organs and tissues that successfully mimic the anatomy and functions of the organ being studied. Tumour cells that have been isolated from cancer patient tissue are converted into an organoid cancer model by use of the extracellular matrix of specific culture media (Voskoglou-Nomikos et al., 2003). Numerous genetic methods can be used to characterize and modify organoids at the cellular and molecular levels, and they can be utilized to find possible causes of cancer. A preliminary genomic, transcriptomic, and metabolic investigation of human cancer organoids opens up new avenues for classifying cancer patients for cytotoxic chemotherapies and targeted therapies. Primary epithelial cell cultures that are embedded in a matrix and continuously proliferate in a manner that is dependent on Wnt signalling and mitogens are known as organoids. Tissue-derived stem cells are implanted into three-dimensional matrix organoids, resulting in self-sufficient structures (Sachs and Clevers, 2014).

PDOs maintain the tumours expression pattern, which includes CNAs, its transcriptional landscape, and its mutation status, as well as sharing structural similarities with the original tumour. PDOs include tumours of the breast, pancreas, stomach, prostate, bladder, and HCC. PDOs often lack essential elements such as blood arteries, immunological cells, and other stromal cells. The lack of immunological elements is the primary barrier to the usage of PDOs in cancer immunotherapy. Because PDO cells primarily exchange materials via low-rate infiltration rather than blood arteries, this has serious consequences for the creation and effectiveness of pharmaceuticals (Huch et al., 2017).

Certain infectious illnesses, such as Epstein-Barr virus, *Helicobacter pylori* in stomach cancer, and *Salmonella enterica* in gallbladder cancer, have a substantial impact on the course of cancer carcinoma. Organoids are cocultured with specific pathogens in order to investigate the mechanism and link between the infectious pathogen and cancer. Studies on stomach organoids have revealed the significant role that chronic *H. pylori* infection plays in the development of gastric cancer. Microinjection of *H. pylori* causes potent primary inflammatory responses due to its capacity to find and colonise the stomach epithelium (Yang et al., 2018). Clonal organoid cultures are grown in healthy organs, and the mutation spectrum unique to each organ may be analysed more easily because of genome sequencing. Utilizing clonal organoid cultures from different parts of the same tumour, intratumour heterogeneity is examined. Organoid cultures are utilized to research mutagenesis processes because of their longer-term genetic stability. Part of the cultural composition of the common cancer organoids is shown in Table 3.

**TABLE 3:** Characteristics of common cancer organoid cultures.

Organoids	Source	Extracellular matrix	Cellular components	Inhibitors	Cell types in organoid	References
Stomach	hPSCs	Matrigel (growth factor reduced)	WNT, FGF, Noggin, Retinoic acid, EGF, ADMEM/F12, penicillin/streptomycin, l-glutamine, B27, N2	A-83-01, Y27632	LGR5 + cells, mucous cells, gastric endocrine cells	Xu et al. (2018), Drost and Clevers (2018)
Prostate	hAdSc	Matrigel (growth factor reduced)	ADMEM, penicillin/streptomycin, primocin, GlutaMAX, B27, EGF, N-acetylcysteine, FGF10, FGF-basic, nicotinamide, testosterone, prostaglandin E2, Noggin, and R-spondin	A-83-01, SB202190	Differentiated CK5 + basal and CK8 + luminal cells	Xu et al. (2018), Drost and Clevers (2018)
Pancreas	hAdSc	Matrigel	ADMEM/F12, penicillin/streptomycin, GlutaMAX, HEPES, B27, N-acetylcysteine, EGF, R-spondin-1, gastrin 1, Wnt3A, Noggin, and FGF	A-83-01	Epithelial ductal cells	Xu et al. (2018), Drost and Clevers (2018)
Liver	hAdSc	Basement membrane extract	Activin A, Wnt, FGF, cAMP, glucocorticoids, ADMEM/F12, penicillin/streptomycin, GlutaMAX, HEPES, B27 (without vitamin A), N2, N-acetylcysteine, nicotinamide, gastrin 1, EGF, FGF10, HGF, forskolin, Rspondin-1, Wnt3A, and Noggin	A-83-01, Y27632	Functional hepatocyte cells	Xu et al. (2018), Drost and Clevers (2018)

Comparing various lesions from the same person helps to understand the mechanisms underlying cancer evolution. Exome sequencing revealed that the lesions all originated from a common source and revealed driver mutations shared by organoids derived from the same person, indicating that these driver mutations occurred before metastatic dissemination. Four independent matched sets of organoid cultures are produced from primary colorectal tumours and metastatic lesions isolated from the same patient (Yang et al., 2018).

Organoids provide a platform for researching the microenvironment around tumours. Malignant cell-tumour microenvironment signalling helps create a favourable habitat for the tumour and offers therapeutic targets that are supportive of it. Stem cells are employed to represent the interactions between cancer cells and other cell types inside the tumour microenvironment because traditional *in vivo* model systems cannot identify the paracrine connections within neoplasm cultures of cancer organoids. Organoids provide a versatile platform for investigating the tumour microenvironment and the complex interactions that support cancer progression. Signaling between malignant cells and components of the tumour microenvironment promotes a supportive niche for tumour growth and represents potential therapeutic targets. Unlike traditional *in vivo* models, organoid cultures allow the study of paracrine interactions between cancer cells and surrounding stromal or immune cells. To model these interactions, stem cell-derived organoids can be co-cultured with other relevant cell types. For example, an ALI system has been developed in which αSMA-positive myofibroblasts and epithelial organoids derived from resected human colorectal cancer tissues are cultured together. While this system preserves the three-dimensional architecture and avoids the selection pressures inherent to monolayer culture, it is limited in its ability to functionally dissect cancer-stromal signaling, as fibroblasts and cancer cells cannot be readily separated for downstream analyses (Tuveson and Clevers, 2019). Organoid-based cancer models have a functional relevance because they can recapitulate the three-dimensional architecture, cellular heterogeneity, genetic profile, and microenvironmental characteristics of patient-derived tumours in a highly physiologically relevant way. Therefore, they provide an excellent platform for translational studies in oncology. Organoid-based cancer models are powerful tools for high-content drug screening and predicting the response to therapeutics, as they facilitate rapid evaluation of the cytotoxic properties of numerous agents, targeted therapies, immunotherapy, and combination regimens in a patient-specific manner. In the area of personalised medicine, organoid-based tumour models provide a critical link between the drug sensitivity profiles from patient-derived samples and the identification of therapeutic strategies for individual patients. Beyond drug screening, organoids are used extensively as models of disease, including modelling of tumour initiation, progression, metastasis, treatment resistance, and the dysregulation of molecular pathways. In addition, organoids are compatible with genomic, transcriptomic, proteomic, and CRISPR-based functional analyses, which enhances their utility in biomarker discovery, target validation, and precision oncology research. As such, organoid models provide an important connection between standard *in vitro* assays and *in vivo* systems and enhance the translational predictability of anticancer drug development (Polak et al., 2024).

## 9 Conclusion and future perspectives

Development of new anticancer drugs depends significantly on reliable preclinical testing methods that evaluate a drug’s effectiveness and safety before it progresses to clinical trials. This process typically follows a stepwise strategy, starting with *in vitro* (test tube or cell culture) studies and moving to *in vivo* animal models. This kind of strategy will assist researchers to establish the most promising drug candidates and minimize the possibility of an ineffective compound being subjected to human testing. The Tetrazolium Salt Assay, Sulphorhodamine B Assay, and Clonogenic Assay are some of the common *in vitro* methods applied to determine the killing or growth inhibitory effect of a given drug on cancer cells. There are higher advanced systems that can be used such as 3D and 4D tumour systems which are more precise and more the way tumours behave within the human body. Nevertheless, *in vitro* models have not yet achieved the whole complexity of the tumour environment and metabolism of drugs and so further experiments need to be done in living animal models. Animal models that involve chemical carcinogens, including the breast cancer induced with DMBA in rats or prostate cancer induced with MNU in gerbils, assist the researcher to study the growth and response of tumours to treatment. These models also entail the knowledge of the way drugs are handled in the body and the side effects they might have. However, they do not completely mirror the differences in genetics and molecules that are observed in human cancers. Lack of a strong correlation between clinical and preclinical outcomes is one of the greatest problems in the development of anticancer drugs. A lot of drugs tested on animals perform well, however, when tested on human beings they fail because of the biological differences. This problem shows the collaboration of more precise models that are more indicative of human cancers.

More recent methods like PDX, organoid cultures, GEMMs have a better predictive capability and may also increase the validity of preclinical studies. To conclude, the existing *in vitro* and *in vivo* models are required in drug development although there are limits that suggest the use of more advanced models. Subsequent attempts must be directed towards developing models that are more similar to human cancers which would be used to bridge the gap between laboratory research and clinical achievement that would eventually result in more effective and safer cancer therapies.

In summary, the current models that are in use, namely, the *in vitro* and the *in vivo* models, though necessary in screening anticancer drugs, have shortcomings that point to the necessity of continued innovation and enhancement. The primary directions of future research should be reducing the rate of translational failures, improving the success rate of the new anticancer therapies, and using more and more complex preclinical models that are highly similar to human cancers. These developments will at some point accelerate the development of safer and more effective cancer medicines and bridge the gap between the preclinical and clinical research. Rather than depending on the outcome of a single experimental system, the development of an anticancer drug requires an integrated multi-model approach, wherein each model provides relevant data from distinct biological and translational levels.

## Glossary

AOM: Azoxymethane
DEN: NNitrosodiethylamine
DiSC: Differentialstainingcytotoxicity
DMBA: Dimethylbenz[a]anthracene
DMSO: Dimethylsulfoxide
EDTA: Ethylenediaminetetraaceticacid
ELISA: Enzyme-linked immunosorbent assay
FACS: Fluorescence-activated cell sorter
FOXO: Forkhead transcription factor
FVB: Friend virus B
GEM: Genetically engineered mouse
HCC: Hepatocellular carcinoma
HPLC: High-Performance Liquid Chromatography
IC: Inhibitory concentration
IMG: Intussusceptive microvascular Growth
LEC: Long-Evans Cinnamon
MCA: Methylcholanthrene
MDR2: Multidrug resistance protein 2
MNU: N-Methyl-N-nitrosourea
MTT: 3-(4,5-Dimethylthiazol-2-yl)-2,5-diphenyltetrazolium Bromide
NAFLD: Non-alcoholic fatty liver disease
NASH: Nonalcoholicsteatohepatitis
NCDS: noncommunicicablediseases
NCI: NationalCancerInstitute
OHBBN: NButylN(4hydroxybutyl)nitrosamine
PCNA: Proliferatingcellnuclearantigen
PDOs: Patientderivedorganoids
PDX: Patientderivedxenografts
PTTG: Pituitarytumourstransforminggene
SBD: StructureBasedDrugDesign
SENCAR: Sensitivetocarcinogenesis
SRB: SulphorhodamineBassay
TPA: Tetradecanoylphorbol-13-acetate
VEGF: Vascular endothelial growth factor
